# Novel Coumarin Derivatives as Potential Urease Inhibitors for Kidney Stone Prevention and Antiulcer Therapy: From Synthesis to In Vivo Evaluation

**DOI:** 10.3390/ph16111552

**Published:** 2023-11-02

**Authors:** Kiran Shahzadi, Syed Majid Bukhari, Asma Zaidi, Tanveer A. Wani, Muhammad Saeed Jan, Seema Zargar, Umer Rashid, Umar Farooq, Aneela Khushal, Sara Khan

**Affiliations:** 1 Department of Chemistry, COMSATS University Islamabad, Abbottabad Campus, Abbottabad 22060, KPK, Pakistan; kiranshahzadi12@gmail.com (K.S.); majidbukhari@cuiatd.edu.pk (S.M.B.); umerrashid@cuiatd.edu.pk (U.R.); umarf@cuiatd.edu.pk (U.F.); aneelakhan460@gmail.com (A.K.); 2 School of Chemistry and Chemical Engineering Technology, Beijing Institute of Technology, Beijing 100811, China; 3 Department of Pharmaceutical Chemistry, College of Pharmacy, King Saud University, P.O. Box 2457, Riyadh 11451, Saudi Arabia; twani@ksu.edu.sa; 4 Department of Pharmacy, The Professional Institute of Health Sciences, Mardan 23200, KPK, Pakistan; 5 Department of Biochemistry, College of Science, King Saud University, P.O. Box 22452, Riyadh 11451, Saudi Arabia; szargar@ksu.edu.sa; 6 Beijing National Laboratory for Molecular Sciences, State Key Laboratory of Molecular Reaction Dynamics, Institute of Chemistry, Chinese Academy of Sciences, Beijing 100190, China

**Keywords:** 4-aminocoumarin, Schiff bases, urease inhibition, molecular docking, antibacterial activity, MD simulation

## Abstract

The presence of ammonium ions in urine, along with basic pH in the presence of urease-producing bacteria, promotes the production of struvite stones. This causes renal malfunction, which is manifested by symptoms such as fever, nausea, vomiting, and blood in the urine. The involvement of urease in stone formation makes it a good target for finding urease enzyme inhibitors, which have the potential to be developed as lead drugs against kidney stones in the future. The documented ethnopharmacology of coumarin 2-one against bacterial, fungal and viral strains encouraged us to synthesize new derivatives of coumarins by reacting aromatic aldehydes with 4-aminocoumarin. The synthesized compounds (**2a** to **11a**) were evaluated for their antimicrobial, in vitro, and in silico properties against the urease enzyme. The study also covers in vivo determination of the synthesized compounds with respect to different types of induced ulcers. The molecular docking study along with extended MD simulations (100 ns each) and MMPBSA study confirmed the potential inhibitory candidates as evident from computed ∆G_bind_ (**3a** = −11.62 and **5a** = −12.08 Kcal/mol) against the urease enzyme. The in silico analyses were augmented by an enzymatic assay, which revealed that compounds **3a** and **5a** had strong inhibitory action, with IC_50_ of 0.412 µM (64.0% inhibition) and 0.322 µM (77.7% inhibition), respectively, compared to standard (Thiourea) with 82% inhibition at 0.14 µM. Moreover, the most active compound, **5a**, was further tested in vivo for antiulcer activity by different types of induced ulcers, including pyloric ligation-, ethanol-, aspirin-, and histamine-induced ulcers. Compound **5a** effectively reduced gastric acidity, lipid peroxidation, and ulceration in a rat model while also inhibiting gastric ATPase activity, which makes it a promising candidate for ulcer treatment. As a result of the current research, **3a** and **5a** may be used as new molecules for developing potent urease inhibitors. Additionally, the compound **3a** showed antibacterial activity against *Staphylococcus aureus* and *Salmonella typhimurium*, with zones of inhibition of 41 ± 0.9 mm and 35 ± 0.9 mm, respectively. Compound **7a** showed antibacterial activity against *Staphylococcus aureus* and *Salmonella typhimurium*, with zones of inhibition of 30 ± 0.8 mm and 42 ± 0.8 mm, respectively. These results prove that the synthesized compounds also possess good antibacterial potential against Gram-positive and Gram-negative bacterial strains.

## 1. Introduction

The development of kidney stones is one of the major health hazards faced by several human beings. The elements for this clinical condition are basic urinary pH with the existence of ammonium ions in the urinary tract and urease-producing bacteria present in the vicinity. Urine formation is known to involve bacterial strains (Gram-positive and Gram-negative), as well as yeast and mycoplasma. Urease present in certain bacterial strains may act as a virulence factor in this scenario. Hence, the combination of phosphate with urine containing magnesium, carbonate apatite, and ammonium leads to the formation of kidney stones. Certain heterocyclic compounds are well known for their antibacterial, antifungal, and antiviral potential. Therefore, the synthesis of new derivatives of such heterocyclic compounds may lead medicinal chemistry towards the discovery of good and cost-effective antimicrobial agents against the drawbacks caused by excessive urease enzyme production [1].

One of the classes of such heterocyclic compounds is coumarin, which belongs to benzopyrans, the leading members of heterocyclic organic compounds. Currently, there is a great emphasis on the synthesis of coumarin-based compounds due to their pharmacological and biological activities. Derivatives of coumarin can be synthesized by using different methods, including Knoevenagel, Kostanecki–Robinson, Perkin, Wittig, Reformatsky, solid-state synthesis of coumarin, and Pechmann reactions [2,3,4].

The 4-hydroxycoumarin anticoagulant is one of the most important derivatives of coumarin. It belongs to the class of anticoagulant (vitamin K antagonist) drug molecules. Said molecule is considerably important, as it can inhibit vitamin K epoxide reductase. It is also of notable interest as being an important reagent for heterocyclization, which will become one of the primary approaches in the forthcoming synthesis of heterocyclic derivatives in the area of combinatorial chemistry [5]. The 4-hydroxycoumarin and its derivatives namely coumatetralyl, acenocoumarol, warfarin, phenprocoumon, and coumachlor show a large biological potential which includes analgesic, anticoagulant, cytotoxic, anti-proliferative, hypnotic and sedative, anti-tumor, anti-inflammatory, antibacterial, antifungal, anticancer, antioxidant and anti-HIV [6,7].

Anti-inflammatory and antioxidant properties of coumarins are known for minimizing the risk of diabetes, cancer, and cardiovascular diseases [8,9,10,11]. Furthermore, the derivatives of coumarin are used for the treatment of renal stones and burns. The inhibition of *5α*-reductase as well as platelet accumulation inhibition has also been reported.

The Schiff base synthesis of coumarins further enhances their biological activities. Schiff bases are obtained by the reaction of amines with a stoichiometric quantity of aldehydes. Schiff bases that contain the azomethine group play an integral role in the field of drug discovery and development and present a large number of pharmacological applications, including anticoagulant, anticonvulsant, antibacterial, antifungal, anti-inflammatory [12,13,14,15], sedative, and antioxidant.

The presence of ammonium ions in urine, along with a basic pH in the presence of urease-producing bacteria, promotes the production of struvite stones. This causes renal malfunction, which is manifested by symptoms such as fever, nausea, vomiting, and blood in the urine. The involvement of urease in stone formation makes it a good target for finding urease enzyme inhibitors, which have the potential to be developed as lead drugs against kidney stones in the future. The documented ethnopharmacology of coumarin 2-one against bacterial, fungal, and viral strains encouraged us to synthesize new derivatives of coumarins by reacting aromatic aldehydes with 4-aminocoumarin. Along with the antibacterial and anti-urease potential of these compounds experimentally, urease inhibition has also been evaluated by an in silico approach followed by the determination of antiulcer properties of the synthesized compounds in different ulcer-induced models in rats.

## 2. Results and Discussion

The synthesis of coumarin derivatives was initially optimized using a range of different conditions, as indicated in Table 1. Following the successful optimization of the initial reaction, we proceeded to synthesize various coumarin derivatives. Additionally, we carried out further optimization for the second step of this process, as outlined in Table 2.

**4**-aminocoumarin

Light yellowish powder; % age yield: 59%; melting point range = 226–227 °C; R_f_ = 0.51 (CH_3_COOC_2_H_5_:CH_3_OH:: 1.5:1); UV-vis (THF) λ_max_ (nm) = 305; FT-IR: ν (cm^−1^) = 3370 (N-H stretching), 1596 (C=O vibration), 3196 (=C-H stretching vibration), 1455 (C=C vibration), 1321 (C-N stretching vibration); ^1^HNMR: (400 MHz, DMSO-*d*_6_); *δ*: 2.51 (s, 2H, H-11), 5.22 (s, 1H, H-9), 7.98 (d, *J* = 8 Hz, 1H, H-6), 7.59 (m, 1H, H-1), 7.31 (t, *J* = 8 Hz, 2H, H-2, 3). ^13^C NMR δ:159.58, 153.47, 152.63, 132.53, 125.74, 122.64, 114.66, 114.45, 86.62.

Schiff bases (**2a**–**11a**)

**Figure 1:**
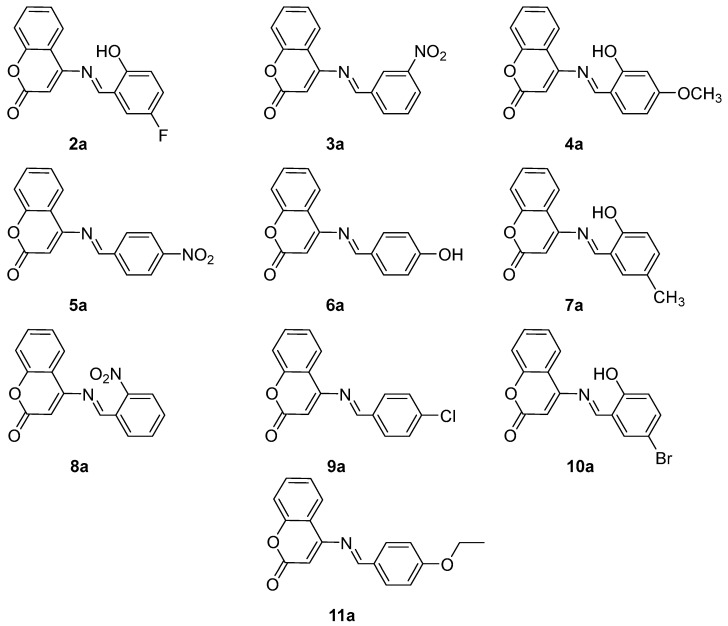
The structures of synthesized derivatives (**2a**–**11a**).

The structures (Figure 1) and spectral data of synthesized derivatives of 4-aminocoumarin are given below.

(*E*)-4-(5-fluoro-2-hydroxybenzylideneamino)-2*H*-chromen-2-one (**2a**):

White solid; yield = 69%; R_f_ = 0.5 (CH_3_COOC_2_H_5_:C_6_H_14_:: 1.5:1); melting point range = 295–298 °C; UV-vis (THF) λ_max_ (nm) = 307; FT-IR: ν (cm^−1^) = 1568 (C=N vibrations), 1701 (C=O stretch), 3048 (=C-H stretching), 1383 (C-F vibration), 3248 (OH vibration); ^1^H-NMR: (400 MHz, CDCl_3_); *δ*: 10.43 (s, 1H, H-13), 5.37 (s, 1H, H-9), 8.16 (d, *J* = 8 Hz, 1H, H-6), 7.53 (m, 1H, H-1), 7.67 (m, 1H, H-2), 8.06 (d, *J* = 8 Hz, 1H, H-3), 7.49 (s, 1H, H-15), 7.03 (m, 1H, H-17), 6.87 (dd, *J* = 8 Hz and 4 Hz, 1H, H-18), 3.51 (s, 1H, H-20). ^13^C NMR δ: 164.44, 164.41, 163.28, 156.23, 156.16, 156.14, 154.45, 154.22, 151.59, 133.22, 128.76, 123.92, 121.28, 121.21, 119.59, 119.43, 118.50, 118.25, 118.18, 117.69, 116.66, 116.50, 102.80

(*E*)-4-(3-nitrobenzylideneamino)-2*H*-chromen-2-one (**3a**):

White powder; yield = 64%; R_f_ = 0.6 (CH_3_COOC_2_H_5_:C_6_H_14_:: 1.5:1); melting point range = 200–202 °C; UV-vis (THF) λ_max_ (nm) = 304; FT-IR: ν (cm^−1^) = 1560 (C=N stretching), 1650 (C=O stretching vibration), 3047 (=C-H vibrations), 1524 and 1341 (NO_2_ stretching), 1443 (C=C, Ar stretching vibrations); ^1^H-NMR: (400 MHz, CDCl_3_); *δ*: 8.10 (s, 1H, H-13), 6.15 (s, 1H, H-9), 7.58 (d, *J* = 8 Hz, 1H, H-6), 7.47 (m, 2H, H-1, 3), 7.54 (t, *J* = 8 Hz, 1H, H-2), 8.02 (d, *J* = 8 Hz, 1H, H-15), 8.12 (d, *J* = 8 Hz, 1H, H-16), 8.17 (d, *J* = 8 Hz, 1H, H-17), 7.69 (s, 1H, H-19). ^13^C NMR δ: 163.28, 163.22, 154.56, 151.39, 146.84, 136.79, 133.55, 130.37, 129.03, 128.60, 123.69, 122.65, 122.13, 118.52, 117.61, 103.03.

(*E*)-4-(2-hydroxy-4-methoxybenzylideneamino)-2*H*-chromen-2-one (**4a**):

Pale-yellow solid; yield = 57%; R_f_ = 0.6 (CH_3_COOC_2_H_5_:C_6_H_14_:: 1.5:1); melting point range = 185–188 °C; UV-vis (THF) λ_max_ (nm) = 368; FT-IR: ν (cm^−1^) = 1543 (C=N stretching), 1596 (C=O vibration), 3061 (=C-H stretching vibration), 3218 (OH stretch), 1453 (C=C vibrations); ^1^H-NMR: (400 MHz, DMSO-*d*_6_); *δ*: 7.33 (s, 1H, H-13), 5.22 (s, 1H, H-9), 7.99 (d, *J* = 8 Hz, 2H, H-6, 15), 7.59 (m, 2H, H-1, 2), 7.30 (d, *J* = 8 Hz, 2H, H-2, 16), 7.38 (s, 1H, H-18), 5.76 (s, 1H, H-20), 3.34 (s, 3H, H-21).

(*E*)-4-(4-nitrobenzylideneamino)-2*H*-chromen-2-one (**5a**):

Off-white solid; yield = 63%; R_f_ = 0.6 (CH_3_COOC_2_H_5_:C_6_H_14_:: 1.5:1); melting point range = 255–257 °C; UV-vis (THF) λ_max_ (nm) = 374; FT-IR: ν (cm^−1^) = 1577 (C=N stretching vibration), 1670 (C=O stretch), 3050 (=C-H vibrations), 1509 and 1340 (NO_2_ stretching vibrations), 1436 (C=C vibrations); ^1^H-NMR: (400 MHz, CDCl_3_); *δ*: 12.54 (s, 1H, H-13), 6.12 (s, 1H, H-9), 8.03 (d, *J* = 8 Hz, 1H, H-6), 7.64 (m, 1H, H-1), 7.69 (t, *J* = 8 Hz, 1H, H-2), 7.73 (d, *J* = 8 Hz, 1H, H-3), 8.17 (d, *J* = 8 Hz, 2H, H-15, 19), 8.19 (d, *J* = 8 Hz, 2H, H-16, 18). ^13^C NMR δ: 164.73, 163.76, 163.28, 158.34, 154.56, 151.48, 133.55, 130.31, 128.60, 123.67, 118.50, 117.55, 114.58, 107.24, 102.80, 101.85, 55.57.

(*E*)-4-(4-hydroxybenzylideneamino)-2*H*-chromen-2-one (**6a**):

White solid; yield = 61%; R_f_ = 0.7 (CH_3_COOC_2_H_5_:C_6_H_14_:: 1.5:1); melting point range = 280–282 °C; UV-vis (THF) λ_max_ (nm) = 305; FT-IR: ν (cm^−1^)= 1582 (C=N stretching vibrations), 1517 (C=O stretching), 3191 (=C-H vibration), 3337 (OH stretching), 1436 (C=C, Ar stretch); ^1^H-NMR: (400 MHz, DMSO-*d*_6_); *δ*: 9.28 (s, 1H, H-13), 4.98 (s, 1H, H-9), 8.51 (d, *J* = 8 Hz, 1H, H-6), 7.46 (t, *J* = 8 Hz, 1H, H-1), 7.68 (t, *J* = 8 Hz, 1H, H-2), 7.41 (d, *J* = 8 Hz, 1H, H-3), 7.11 (d, *J* = 0 Hz, 2H, H-15, 19), 6.64 (d, *J* = 8 Hz, 2H, H-16, 18), 9.71 (br.s, 1H, H-20, -OH). ^13^C NMR δ: 164.05, 163.28, 154.56, 151.58, 148.24, 139.10, 133.55, 128.60, 128.41, 124.30, 123.69, 118.52, 117.61, 103.03.

(*E*)-4-(2-hydroxy-5-methylbenzylideneamino)-2*H*-chromen-2-one (**7a**):

Yellow solid; yield = 79%; R_f_ = 0.6 (CH_3_COOC_2_H_5_:C_6_H_14_:: 1.5:1); melting point range = 253–255 °C; UV-vis (THF) λ_max_ (nm) = 368; FT-IR: ν (cm^−1^) = 1545 (C=N stretch), 1608 (C=O stretching vibration), 3150 (=C-H stretching), 3374 (OH stretching vibrations), 2959 (C-H vibration), 1454 (C=C, Ar stretching vibration); ^1^H-NMR: (400 MHz, DMSO-*d*_6_); *δ*: 10.22 (s, 1H, H-13), 5.22 (s, 1H, H-9), 7.99 (d, *J* = 8 Hz, 1H, H-6), 7.33 (m, 1H, H-1), 7.59 (t, *J* = 8 Hz, 1H, H-2), 6.90 (d, *J* = 8 Hz, 1H, H-3), 7.31 (s, 1H, H-15), 7.45 (d, *J* = 8 Hz, 1H, H-17), 7.35 (d, *J* = 8 Hz, 1H, H-18), 7.38 (br.s, 1H, H-20, -OH), 2.24 (s, 3H, H-21, -CH_3_). ^13^C NMR δ: 164.31, 163.28, 157.14, 154.45, 151.59, 133.27, 132.49, 131.98, 129.44, 128.76, 123.92, 120.58, 118.50, 117.69, 116.51, 102.80, 20.61.

(*E*)-4-(2-nitrobenzylideneamino)-2*H*-chromen-2-one (**8a**):

White solid; yield = 60%; R_f_ = 0.6 (CH_3_COOC_2_H_5_:C_6_H_14_:: 1.5:1); melting point range = 265–268 °C; UV-vis (THF) λ_max_ (nm) = 304; FT-IR: ν (cm^−1^) = 1571 (C=N stretching vibrations), 1667 (C=O vibration), 3249 (=C-H stretching), 1517 and 1348 (NO_2_ stretching vibrations), 1439 (C=C, Ar stretching), ^1^H-NMR: (400 MHz, DMSO-*d*_6_); *δ*: 8.88 (s, 1H, H-13), 6.49 (s, 1H, H-9), 7.75 (d, *J* = 8 Hz, 1H, H-6), 7.40–7.46 (m, 2H, H-1,3), 7.50 (t, *J* = 8 Hz, 1H, H-2), 7.92 (d, *J* = 8 Hz, 1H, H-15), 7.66 (t, *J* = 8 Hz, 1H, H-16), 7.62 (m, 1H, H-17), 8.12 (d, *J* = 8 Hz, 1H, H-18). ^13^C NMR δ: 163.28, 159.16, 154.56, 151.06, 147.42, 133.55, 131.35, 130.56, 130.00, 128.60, 128.03, 125.27, 123.69, 118.53, 117.61, 102.80.

(*E*)-4-(4-chlorobenzylideneamino)-2*H*-chromen-2-one (**9a**):

Yellowish powder; yield = 57%; R_f_ = 0.6 (CH_3_COOC_2_H_5_:C_6_H_14_:: 1.5:1); melting point range = 255–257 °C; UV-vis (THF) λ_max_ (nm) = 301; FT-IR: ν (cm^−1^) = 1524 (C=N stretch), 1669 (C=O vibrations), 3047 (=C-H stretching vibration), 1437 (C=C vibrations); ^1^H-NMR: (400 MHz, CDCl_3_); *δ*: 11.34 (s, 1H, H-13), 6.07 (s, 1H, H-9), 8.08 (m, 1H, H-6), 7.44 (d, *J* = 8 Hz, 2H, H-1, 3), 7.66 (t, *J* = 8 Hz, 1H, H-2), 7.32 (d, *J* = 8 Hz, 2H, H-15, 19), 7.18 (d, *J* = 8 Hz, 2H, H-16, 18). ^13^C NMR δ: 164.10, 163.28, 154.30, 151.72, 135.21, 133.39, 133.26, 129.32, 128.92, 128.76, 123.92, 118.56, 117.69, 103.16.

(*E*)-4-(5-bromo-2-hydroxybenzylideneamino)-2*H*-chromen-2-one (**10a**):

White solid; yield = 75%; R_f_ = 0.6 (CH_3_COOC_2_H_5_:C_6_H_14_:: 1.5:1); melting point range = 276–278 °C; UV-vis (THF) λ_max_ (nm) = 321; FT-IR: ν (cm^−1^) = 1524 (C=N stretching vibration), 3342 (O-H stretching vibration), 1670 (C=O stretching vibration), 3208 (=C-H stretching vibration), 1509 (C=C, Ar stretching vibration); ^1^H-NMR: (400 MHz, DMSO-*d*_6_); *δ*: 8.18 (s, 1H, H-13), 5.54 (s, 1H, H-9), 8.08 (d, *J* = 8 Hz, 1H, H-6), 7.56 (t, *J* = 8 Hz, 1H, H-1), 7.69 (t, *J* = 8 Hz, 1H, H-2), 7.36 (t, *J* = 8 Hz, 1H, H-3), 7.43 (s, 1H, H-15), 7.31 (d, *J* = 8 Hz, 1H, H-17), 7.22 (d, *J* = 8 Hz, 1H, H-18), 7.85 (br.s, 1H, H-20, -OH). ^13^C NMR δ: 164.29, 163.28, 157.95, 154.56, 151.59, 136.03, 133.25, 133.20, 128.61, 123.83, 120.65, 118.50, 117.93, 117.80, 112.47, 102.94.

(*E*)-4-(4-ethoxybenzylideneamino)-2*H*-chromen-2-one (**11a**):

White powder; yield = 69%; R_f_ = 0.6 (ethyl acetate:*n*-hexane:: 1.5:1); m.p. = 264–266 °C; UV-vis (DCM) λ_max_ (nm) = 305 nm; FT-IR:ν = 1509 cm^−1^ (C=N stretching vibration), 1650 cm^−1^ (C=O stretching vibration), 3125 cm^−1^ (=C-H stretching vibration), 1443 cm^−1^ (C=C, Ar stretching vibration), 1099 cm^−1^ (C-O stretching vibration); ^1^H-NMR: (400 MHz, DMSO-*d*_6_); *δ*: 9.7 (s, 1H, H-13), 5.04 (s, 1H, H-9), 8.54 (d, *J* = 8 Hz, 1H, H-6), 7.50 (t, *J* = 8 Hz, 1H, H-1), 7.67 (t, *J* = 8 Hz, 1H, H-2), 7.45 (d, *J* = 8 Hz, 1H, H-3), 7.21 (d, *J* = 8 Hz, 2H, H-15, 19), 6.79 (d, *J* = 8 Hz, 2H, H-16, 18), 3.92 (q, *J* = 8 Hz, 2H, H-20), 1.26 (t, *J* = 8 Hz, 3H, H-21). ^13^C NMR δ: 164.14, 163.28, 160.88, 154.56, 151.58, 133.55, 130.24, 129.16, 128.60, 123.69, 118.52, 117.61, 114.73, 103.03, 63.56, 14.67.

### 2.1. UV-Vis Analysis

Absorbance spectra of all the newly synthesized Schiff bases under investigation were recorded from 200 nm to 800 nm using a quartz cuvette (10 mm), and THF was used as solvent. All the newly synthesized compounds possess a coumarin ring in their chemical structure. The maximum absorbance of compound **2a** was recorded at 305 nm, whereas spectra for compounds **3a**–**11a** showed maximum absorbance at 307, 304, 368, 305, 374, 368, 304, 310, 301, 321, and 305 nm, respectively. The compounds **4a**, **7a**, and **8a** showed a blue shift (hypsochromic), whereas the compounds **3a**, **5a**, **6a**, **9a**, **10a**, and **11a** showed a red shift (bathochromic) compared to the starting material (compound **2**). The shift in maximum absorbance can be attributed to extended conjugation and provides evidence in favor of the successful synthesis of the anticipated products.

### 2.2. FT-IR Analysis

The FT-IR spectrum of newly synthesized compound **2a** shows a characteristic absorption peak at 3370 cm^−1^ with a shoulder band at 3484 cm^−1^, which corresponds to the primary amine group (N-H). These characteristic bands disappear in the FT-IR spectra of synthesized Schiff bases. Also, a characteristic absorption peak at around 1517–1575 cm^−1^ appears in each FT-IR spectrum of Schiff bases, which corresponds to strong C=N vibrations [16]. This analysis further confirms that the amino group of compound **2a** has successfully been converted to an imine group in each succeeding Schiff base compound. Furthermore, the FT-IR spectra of newly synthesized compounds exhibit a strong absorption peak at 3047–3214 cm^−1^, which can be attributed to the presence of a methylene group (=C-H) [17]. The appearance of this particular signal indicates that this moiety did not get involved in the reaction with substituted aromatic aldehydes (Figure S1).

### 2.3. NMR Spectral Analysis

The NMR spectra of newly synthesized compounds were obtained using a Bruker AM-400 MHz NMR spectrometer. All of the compounds were either soluble in DMSO-d_6_ or CDCl_3_. The ^1^H-NMR data of all the newly synthesized compounds confirmed their proposed structures.

In the case of compound **2a**, a singlet appearing at *δ* 2.51 ppm accounted for the two protons of the amino group (NH_2_). The active methylene proton in compound **2a** at position 9 has no neighboring proton; therefore, it showed a singlet at *δ* 5.22 ppm. The proton at position 6 shows strong coupling with its ortho proton, does not show long-range coupling, and gives a doublet signal at *δ* 7.98 ppm. The proton at position 1 shows a multiplet signal at *δ* 7.59 ppm, which is due to a long-range coupling. Protons at positions 2 and 3 are neighboring protons and show a triplet signal at *δ* 7.31 ppm. All of these signals confirm the successful synthesis of compound **2a** (4-aminocoumarin).

The singlet peak appearing at *δ* 2.51 ppm due to two protons of the amino group (NH_2_) in 4-aminocoumarin disappears in the ^1^H-NMR spectrum of Schiff bases (**2a**–**11a**); instead, a singlet due to azomethine proton (N=CH) appears at 7.43–12.5 ppm. The shielding and deshielding of the imine proton are due to the mesomeric effect of pi-electrons of aromatic and aldehydic moiety. The appearance of a characteristic singlet peak due to methylene proton at *δ* 4.98–6.49 ppm in the ^1^H-NMR spectra is strong evidence for the formation of Schiff bases (Figure S1).

### 2.4. Antibacterial Activity

**Figure 2:**
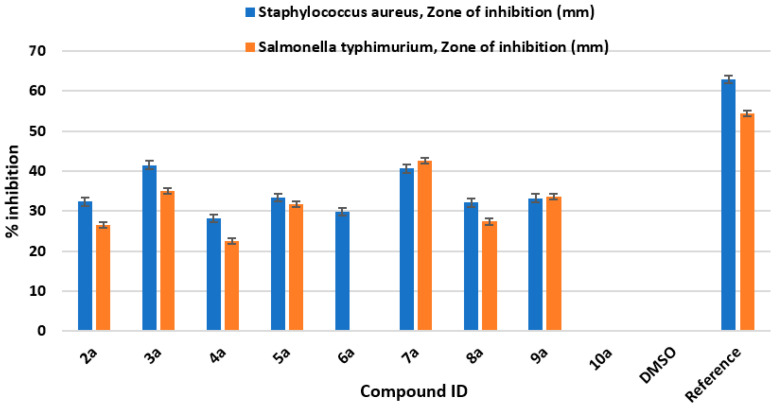
Antibacterial bioassay screening of newly synthesized derivatives (**2a**–**11a**).

All the newly synthesized Schiff bases of 4-aminocoumarin (**2a**–**11a**) were tested against two bacterial strains: *Salmonella typhimurium* (Gram-negative) and *Staphylococcus aureus* (Gram-positive). Zones of inhibition (mm) of all the synthesized compounds against these two strains are shown in Figure 2.

The zones of inhibition were measured in millimeters. The 50 μg/mL concentration of ciprofloxacin (reference drug) showed 62.9 ± 0.94 and 54.4 ± 0.50 mm zones of inhibition against *Staphylococcus aureus* and *Salmonella typhimurium*, respectively. The negative control (DMSO) did not show any activity against these two strains. The synthesized compounds were also tested against the two bacterial strains (concentration: 200 μg/mL). The compounds **3a** and **7a** showed higher zones of inhibition against these two bacterial strains compared to other synthesized derivatives. Compound **3a** showed antimicrobial activity against *Staphylococcus aureus* with a zone of inhibition of 41.5 ± 0.96 mm and against *Salmonella typhimurium* with a zone of inhibition 35 ± 0.90. Also, compound **7a** showed antibacterial activity against *Staphylococcus aureus* with a zone of inhibition of 40.6 ± 0.76 mm and against *Salmonella typhimurium* with a zone of inhibition of 42.6 ± 0.76 mm. Hence, it can be deduced that compound **3a** is highly active against *Staphylococcus aureus* compared to compound **7a**, whereas compound **7a** is comparatively more effective against *Salmonella typhimurium* compared to compound **3a**. The antibacterial bioassay proves that compound **3a** can be used as a lead for the development of antibacterial drugs against *Staphylococcus aureus* and compound **7a** can be taken as an antibiotic against the Gram-negative bacterial strain *Salmonella typhimurium*.

### 2.5. In Vitro Urease Inhibition

In continuation of the in silico studies, compounds **3a** and **5a** were subjected to in vitro urease inhibition assay. The results of this assay further confirm the findings obtained as a result of in silico studies. The inhibition potential of compounds **3a** and **5a** is comparable to that of the standard used (thiourea). Further optimization of **3a** and **5a** may lead to the development of new standards for future use in urease inhibition assays (Table 3).

### 2.6. In Silico Studies

Urease has the tendency to catalyze the hydrolysis of urea into ammonia and carbon dioxide. It is a key virulence factor for a wide range of human infections: 10 of the 12 antibiotic-resistant priority pathogens designated by the World Health Organization (WHO) in 2017 are ureolytic bacteria that colonize and thrive in the host organism using urease activity [1,18].

All of the synthesized compounds were screened against a minimized crystal structure and the active site was identified by using the site finder tool embedded in MOE; the rest of the parameters were kept as default, with each molecule sampled in 10 conformations. All of the compounds docked into the urease’s active pocket showed a strong binding affinity with the active site residues which is evident from ∆G_bind_ score (Table 4). All of the compounds have molecular weights within 300–350 and calculated logP values of 0.3–3.2, which places them within the lead-like range. Although all reported compounds are topologically similar to one another, binding affinity and docked poses suggest that compounds **3a** (−8.67 Kcal/mol), **5a** (−7.44 Kcal/mol) and **9a** (−6.96 Kcal/mol) fall into the best candidates for activity against the urease enzyme. In the docked poses, all the compounds showed van der Waal interaction as well as H–arene interaction through an unprecedented coumarin functional group.

**Figure 3:**
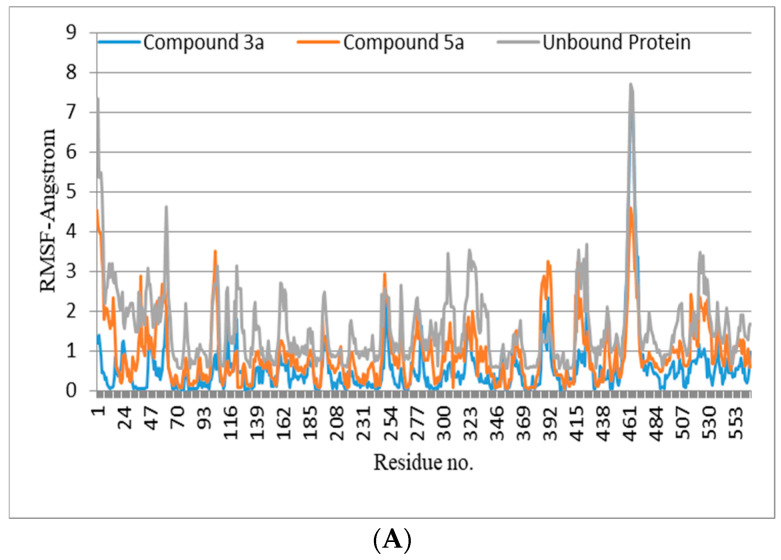

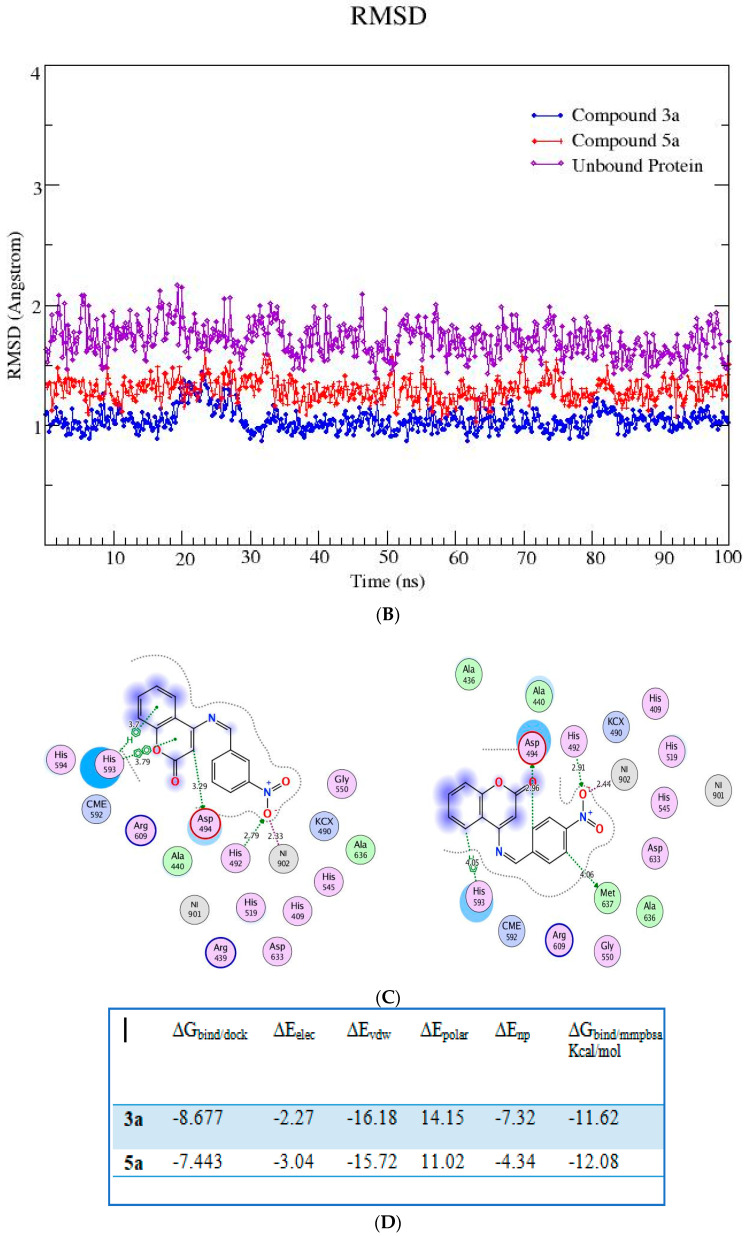

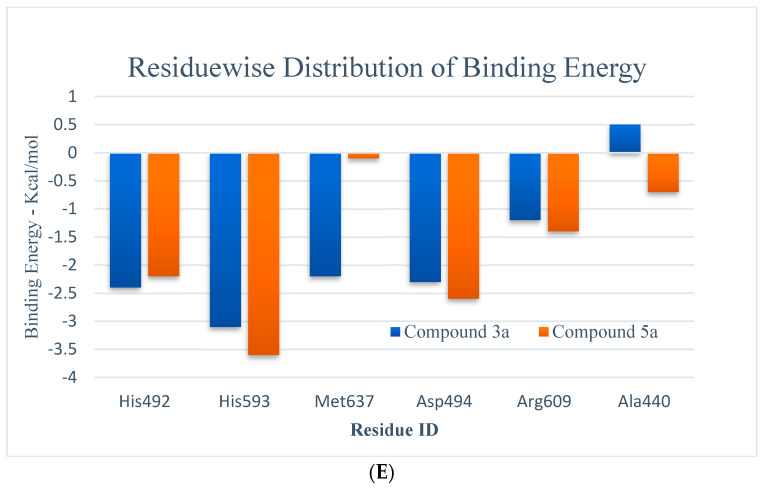
(**A**,**B**) RMSD (all-atom) and RMSF (per residue) plot of urease enzyme in free and bound form with respect to initial minimized structure. Protein—ligand complexes are color—coded as **3a** (blue), **5a** (red), and free protein (violet). (**C**) 2D interaction plots of **3a** and **5a** with urease protein using LigX tools of MOE software (https://www.chemcomp.com/Products.htm. The three-letter amino acid code is assigned to each residue. (**D**) Free energy of binding ΔG_bind_ for **3a** and **5a** compounds using molecular docking as well as an MMPBSA approach. (**E**) Major residues contributing toward protein—ligand interactions. (All values are given in Kcal/mol).

The lead compounds identified as a result of the molecular docking study were subjected to MD simulations in order to understand the structural dynamics, which is essential for identifying potential inhibitors associated with protein inhibition mechanisms. For all trajectories, RMSD calculations were performed to investigate the time-evolved behavior of the protein. Amplitude perturbations of about >2Å were detected in free protein, thereby illustrating the complicated structural changes involved with protein expansion over time. The bound protein has reduced dynamics relative to the free protein, as evidenced by the RMSD result. The average RMSD for all **3a** and **5a** complexed proteins revealed dynamics ranging from 1.2 to 1.4 Å. The attachment of **3a** and **5a** in the active site shifts the protein to a more rigid texture, whereas the free protein is more flexible in its motions. The RMSF is used to analyze the flexibility of individual residues in bound and free proteins. The orientation of the helix-turn-helix motif has a significant impact on urease enzyme binding to compounds (Figure 3A).

The RMSFs of ligand-bound urease are lower than those of free protein, and they are more prominent in the active site. An MMPBSA study was carried out to determine the thermodynamic parameters of the protein–ligand complexes, such as binding free energy and van der Waals, electrostatic, and polar solvation energies for the last 50 ns of the MD simulation trajectories. The binding energy of **3a** and **5a** bound urease complexes remained stable during the explored time scale, as evidenced by ∆G_bind_.

The MMPBSA data analysis aids in calculating the contribution of individual amino acid residues to the total binding energy. The binding of both compounds is adjacent to the nickel ion, in a similar fashion to that of either urea or thiourea fragment which were being complexed by nickel (II) ion. The determination of trajectories that were gained also exposes that binding of **3a** and **5a** to the target protein occurs. It also shows effectively that the compounds under consideration efficiently take up the active sites of the target protein. It has also been observed that tight attachment of the helix-turn-helix motif occurs as a cover on the active site space. This results in obstruction of the flap closure of the urease active site and consequently leads towards inhibition of the urease enzyme. The studies of interaction showed that binding of the sample compounds within the active pocket leads towards the generation of Ni-O electrostatic bonds, whereby nickel-bound oxygen of the compounds **3a** and **5a** forms H-bonds with His492. Apart from this—the key residues (Arg609, Asp494, Arg439, Met697) facilitating the binding of the compounds **3a** and **5a** to the active site residues—a group of histidines (His593, His594, His492) encapsulated the active site, forming a highly charged atmosphere. Ni atom facilitates the binding of the substrate (urea) to the active site; however, the compounds **3a** and **5a** block the entry of the substrate via strongly coordinating with metal at one end and histidine at the other end. The formation of H-bonds between compounds **3a** and **5a** to that of active-site charged residues act as an additional force confirming the strong binding of compounds inside the active pocket.

### 2.7. In Vivo Pharmacology

#### 2.7.1. Pylorus Ligation Activity

**Figure 4:**
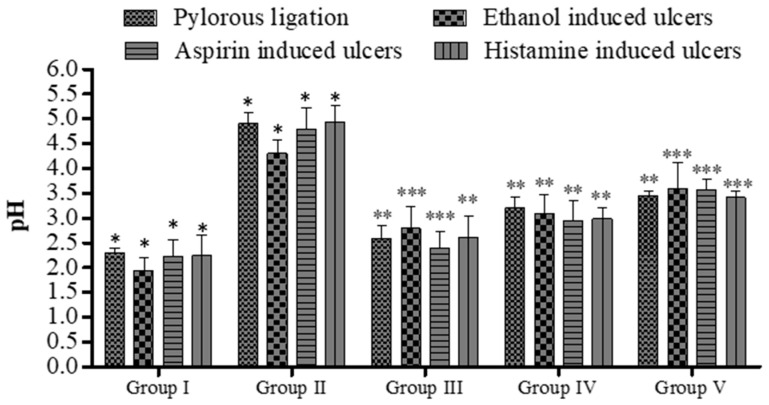
Effect of **5a** on pH of gastric contents in rats. Data are shown as mean ± SEM (*n* = 6). Group II was compared with groups III–V; * *p* < 0.05, ** *p* < 0.01; *** *p* < 0.001.

**Figure 5:**
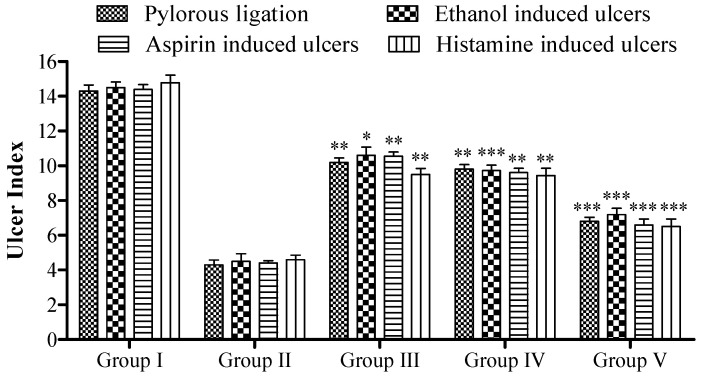
Effect of **5a** on rat’s ulcer index. Data are shown as mean ± SEM (*n* = 6). Group II was compared with groups III–V; * *p* < 0.05, ** *p* < 0.01; *** *p* < 0.001.

**Figure 6:**
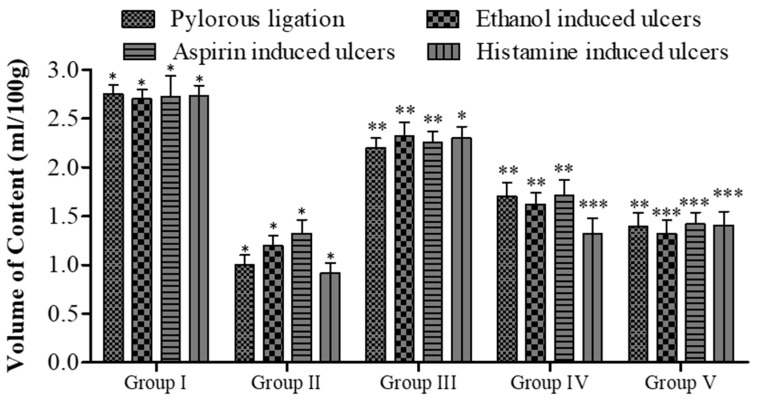
Effect of **5a** on gastric contents volume in rats. Data are shown as mean ± SEM (*n* = 6). Group II was compared with groups III–V; * *p* < 0.05, ** *p* < 0.01; *** *p* < 0.001.

**Figure 7:**
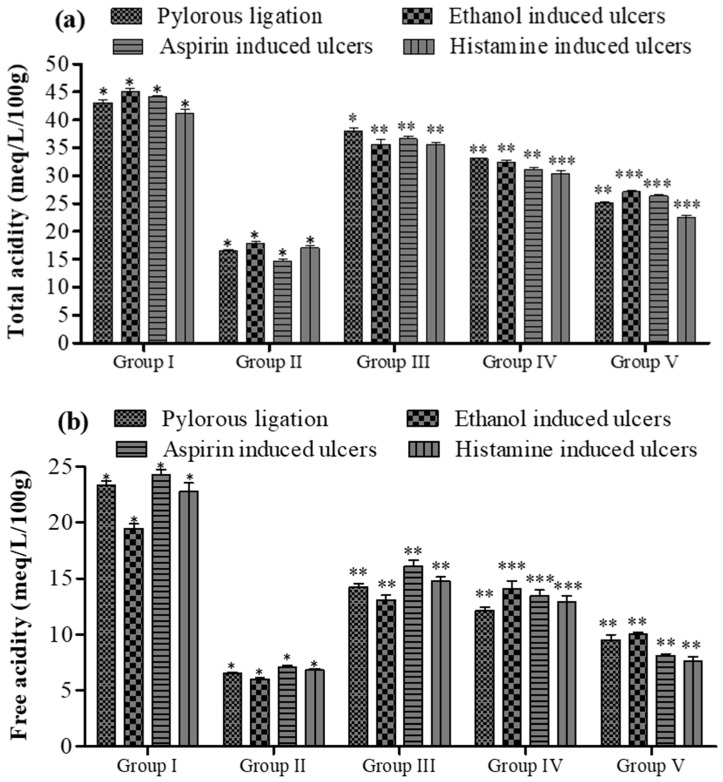
Effect of **5a** on (**a**) total and (**b**) free acidity of gastric contents in rats. Data are shown as mean ± SEM (*n* = 6). Group II was compared with groups III–V; * *p* < 0.05, ** *p* < 0.01; *** *p* < 0.001.

The pyloric ligation ulcer was induced in the rat’s stomach, and the stomach was ligated for approximately 6 h. The insertion of compound **5a** resulted in a reduction in the ulcer index (14.30 ± 0.34 vs. 6.80 ± 0.24, Group I vs. Group V; see Figure 4). In terms of pH, fewer changes were observed (Figure 5), while the gastric content volume after the administration of **5a** was 2.75 ± 0.10 vs. 1.40 ± 0.14 (Group I vs. Group V; Figure 4). The total acidity was 43.10 ± 0.52 meq/L/100 g vs. 25.10 ± 0.16 meq per liter per 100 g (Group I vs. Group V; Figure 6), while the free acidity was 23.34 ± 0.42 meq per liter per 100 g vs. 9.50 ± 0.46 meq per liter per 100 g (Group I vs. Group V; Figure 6), and both were reduced upon the insertion of compound **5a**. Similarly, lipid peroxidation (0.70 ± 0.02; Group I) was reduced (0.42 ± 0.046; Group V, *** *p* < 0.001; Figure 7) upon the administration of compound **5a**.

#### 2.7.2. Ethanol-Induced Ulcer

**Figure 8:**
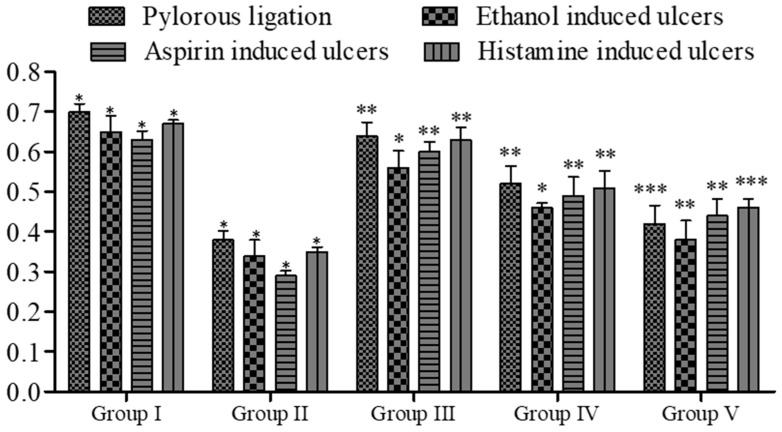
Effect of **5a** on lipid peroxidation of gastric contents in rats. Data are shown as mean ± SEM (*n* = 6). Group II was compared with groups III–V; * *p* < 0.05, ** *p* < 0.01; *** *p* < 0.001.

To investigate ethanol-induced ulcers in the rat model, we administered compound **5a**, which exhibited promising results by significantly decreasing the ulcer index (*** *p* < 0.001; 14.50 ± 0.32 vs. 7.20 ± 0.36, Group I vs. Group V; see Figure 5). Gastric content pH is shown in Figure 4. Similarly, the gastric content volume (** *p* < 0.01) decreased after the insertion of the potent compound **5a** (2.70 ± 0.10 vs. 1.32 ± 0.14, Group I vs. Group V; Figure 6). The pH of the gastric contents (** *p* < 0.01, Group V) also increased (1.94 ± 0.26 in Group I, 3.60 ± 0.52 in Group V). The total acidity, initially at 45.10 ± 0.52 meq per liter per 100 g (Group I), was reduced to 27.10 ± 0.28 meq per liter per 100 g (Group V, *** *p* < 0.001; Figure 7), while the free acidity levels (19.45 ± 0.43 meq per liter per 100 g; Group I; Figure 8) decreased to 10.01 ± 0.16 meq per liter per 100 g (Group V, ** *p* < 0.01). Similarly, the rate of malondialdehyde formation (0.65 ± 0.040; Group I) was reduced to 0.38 ± 0.048 (Group V, *** *p* < 0.001; Figure 7).

Conclusively, the administration of compound **5a** demonstrated promising results in reducing ethanol-induced ulcers in a rat model, as indicated by a significant decrease in the ulcer index and alterations in gastric pH, content volume, total acidity, free acidity, and malondialdehyde formation. These findings suggest the potential therapeutic value of compound **5a** for ulcer treatment.

#### 2.7.3. Aspirin-Induced Ulcer

In the dose range of 20 and 40 mg/kg, compound **5a** demonstrated a significant reduction in the ulcer index (*** *p* < 0.001; 9.60 ± 0.26 vs. 14.40 ± 0.28, Group IV vs. Group I; 6.60 ± 0.34 vs. 14.40 ± 0.28, Group V vs. Group I; see Figure 5) in rats treated with aspirin, along with pH alterations (Figure 4). Similarly, compound **5a** elevated gastric pH from 2.22 ± 0.34 (Group I) to 2.94 ± 0.42 (Group IV, ** *p* < 0.01) and 3.58 ± 0.22 (Group V, *** *p* < 0.001). A significant decrease in gastric content volume was observed with the treatment of compound **5a** (** *p* < 0.01; Group IV and Group V; Figure 6). Total acidity, initially at 44.23 ± 0.17 meq per liter per 100 g (Group I), decreased to 26.34 ± 0.34 meq per liter per 100 g (Group V, *** *p* < 0.001; Figure 7), while free acidity values decreased from 24.23 ± 0.53 meq per liter per 100 g (Group I) to 8.10 ± 1.16 meq per liter per 100 g (Group V, ** *p* < 0.01; Figure 6). Correspondingly, the rate of malondialdehyde formation decreased from 0.63 ± 0.022 (Group I) to 0.44 ± 0.042 (Group V, *** *p* < 0.001; Figure 8).

#### 2.7.4. Histamine-Induced Ulcer

Administration of histamine was shown to be ulcerogenic in the rats. The ulcer index in the group I animals was (14.78 ± 0.44; Figure 5). Compound **5a** was administered, and displayed a decline in ulcer severity and ulcer index (9.44 ± 0.42 in Group IV, ** *p* < 0.01; 6.52 ± 0.42 in Group V, *** *p* < 0.001; Figure 3). The pH of the gastric content (2.24 ± 0.42 in Group I; Figure 4) was elevated (2.62 ± 0.42 in Group III, ** *p* < 0.01; 2.98 ± 0.22 in Group IV, ** *p* < 0.01; 3.42 ± 0.12 in Group V, *** *p* < 0.001; Figure 4). A noteworthy decline in the gastric contents volume was observed (2.73 ± 0.11 versus 1.41 ± 0.14., Group I versus Group V; *** *p* < 0.001 in Group V; Figure 6). The total acidity was 41.24 ± 0.66 meq per liter per 100 g (Group I) and reduced to 22.46 ± 0.42 meq per liter per 100 g (Group V, *** *p* < 0.001; Figure 5), while the free acidity was 22.78 ± 0.78 meq per liter per 100 g (Group I; Figure 7) decreased to 7.60 ± 0.44 meq per liter per 100 g (Group V, ** *p* < 0.01). Likewise, the rate of the formation of the malondialdehyde (0.67 ± 0.010; Group I) was reduced (0.46 ± 0.022; Group V, *** *p* < 0.001; Figure 7).

#### 2.7.5. H^+^–K^+^ ATPase Assay

**Figure 9:**
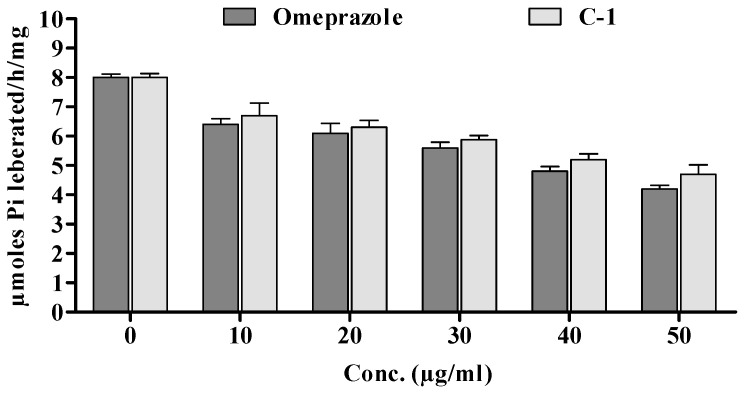
Effect of in vitro enzymatic studies of omeprazole and **5a**.

Compound **5a** was also demonstrated to significantly (* *p* < 0.05) affect gastric mucosal homogenate in rats. Its inhibitory potential was concentration-dependent, and the tested drug showed a comparable effect to omeprazole. Specifically, compound **5a** significantly reduced the hydrolysis of ATP (see Figure 9) through gastric ATPase, with an IC_50_ of 30 μg/mL, which was very similar to the IC_50_ of omeprazole, used as a positive control (IC_50_ of 22 μg/mL).

In the rat model with pyloric ligation-induced ulcers, the animal stomachs were ligated for approximately 6 h. This resulted in a notable increase in total acidity (43.10 ± 0.52 meq/L/100 g vs. 25.10 ± 0.16 meq per liter per 100 g) and free acidity (23.34 ± 0.42 meq per liter per 100 g vs. 9.50 ± 0.46 meq per liter per 100 g) in Group I compared to Group V (as shown in Figure 7b). However, upon the introduction of compound **5a**, these acidity levels were significantly reduced.

Likewise, lipid peroxidation, which was at 0.70 ± 0.02 in Group I, showed a significant reduction (0.42 ± 0.046) in Group V upon the administration of compound **5a**. This suggests the potential effectiveness of compound **5a** in reducing ulceration induced by ethanol in the rat model. Furthermore, the gastric content pH, as depicted in Figure 4, was notably affected. The insertion of compound **5a** resulted in a significant increase in gastric pH from 2.22 ± 0.34 (Group I) to 2.94 ± 0.42 (Group IV, ** *p* < 0.01) and 3.58 ± 0.22 (Group V, *** *p* < 0.001). Additionally, the volume of gastric content decreased significantly with the treatment of compound **5a**. Moreover, compound **5a** demonstrated a significant reduction in the hydrolysis of ATP, as shown in Figure 9, with an IC_50_ of 30 μg/mL, which is comparable to the positive control omeprazole (IC_50_ of 22 μg/mL). This suggests the potential of compound **5a** as a gastric ATPase inhibitor.

Purity of the synthesized compounds

The purity of two best-acting derivatives was confirmed using HPLC (Figure S1, Compound **3a** and **5a**).

## 3. Materials and Methods

### 3.1. Chemicals and Reagents

The ammonium acetate, 4-hydroxycoumarin, substituted aldehydes, methanol, and ethanol were acquired from Sigma Aldrich/Fluka. All of the purchased chemicals were used as acquired, as they were of analytical grade; no purifying methods were applied to the purchased chemicals. TLC plates (silica gel 60 F^254^) used in this study were manufactured by MERCK^®^. Melting point determination of synthesized compounds was carried out by using the Stuart digital melting point apparatus SMP_10_. The UV-vis analysis was performed on a PG Instruments T80 + UV-vis spectrometer (solvent: THF) using a 10 mm quartz cuvette. Fourier-transform infrared spectral analysis was carried out on a PerkinElmer spectrum 100 FT-IR spectrometer. The proton nuclear magnetic resonance spectroscopic analysis δ (ppm) was conducted in chloroform–DMSO-*d*_6_ on a Bruker AM-400 MHz NMR spectrometer. The antimicrobial potential of synthesized derivatives was evaluated by means of the agar well diffusion method. The urease inhibition potential of these newly synthesized compounds was also evaluated on SpectraMax M2 by the indophenol method.

### 3.2. Experimental Synthesis of 4-Aminocoumarin and Its Schiff Base Derivatives

The analogue synthesis of 4-hydroxycoumarin was performed by following a two-step mechanism. In the 1st step, the synthesis of 4-aminocoumarin was carried out under solvent-free conditions as per reported literature [19]. With this method, 4-hydroxycoumarin (1.07 g; 6.6 mmol) and ammonium acetate (7.87 g; 0.1 mol) were melted at 170 °C for 12 h, followed by stirring at constant speed for 3 h at ambient temperature [20]. The mixture was left to cool down at room temperature. The reaction completion was checked on thin-layer chromatography (*n*-hexane: ethyl acetate: 1:1.5). Water was added to the reaction mixture on reaction completion, followed by filtration.

**Scheme 1:**
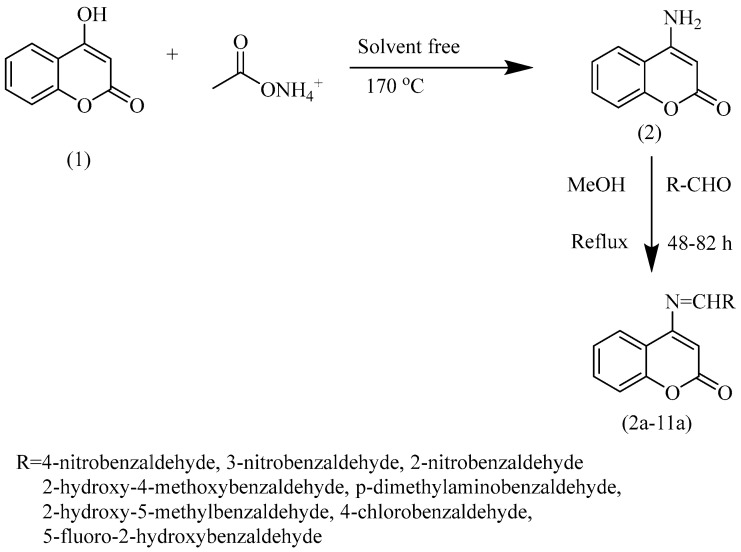
Synthetic scheme presenting two-step synthesis of 4—aminocoumarin and its derivatives. Step (1) requires 170 °C and is a solvent-free synthetic step. Step (2) is the addition of selected aldehyde under reflux conditions using methanol as solvent. This step requires a temperature ranging from 48 to 82 °C, depending upon the aldehyde used.

In the next step, Schiff bases of 4-aminocoumarin were synthesized by condensing a stoichiometric amount of 4-aminocoumarin (1.0 mmol) with substituted aromatic aldehydes (1.0 mmol) in the presence of dry distilled methanol. Refluxing of the mixture was carried out for 48–72 h depending upon the aldehyde used. The reaction completion was checked on thin-layer chromatography (*n*-hexane: ethyl acetate: 1:1.5). Acetic acid was added as a catalyst in small quantities. After the reaction, the product was precipitated and subsequently filtered, followed by its washing using chilled methanol to obtain pure product with moderate-to-good yield. The reactions take a long time to complete, though. The first step takes 14 to 15 h to complete, whereas, depending upon the aldehyde chosen, the second step may complete from 48 to 82 h, thereby giving 65% to 75% yields of resultant products (Scheme 1).

### 3.3. Antibacterial Activity

Antibacterial activity of the synthesized compounds was assessed using the agar well diffusion method. Two bacterial strains selected for this experiment were *Salmonella typhimurium* and *Staphylococcus aureus*. The former is a round-shaped bacterial strain that is Gram-positive and has the tendency to cause food poisoning, skin infections and respiratory tract infections. The latter is a rod-shaped Gram-negative bacterial strain capable of causing typhoid fever, weakness, stomach pain, headache and loss of appetite. Some compounds show best inhibition potential, as their zone of inhibition (mm) is less than the standard drug used.

### 3.4. Urease Inhibition Assay

The presence of *H. pylori* in the stomach has been associated with a range of gastric disorders, including peptic ulcers, gastritis, and an increased risk of gastric cancer. The bacterium’s ability to manipulate the gastric environment through urease activity underscores its role in the development and progression of these conditions. Different studies have targeted *H. pylori* and its urease activity as potential avenues for therapeutic intervention and disease management. The evaluation of urease inhibition potential of the compounds under discussion was carried out by the indophenol method. In this method, the production of NH_3_ occurs in situ and is measured as a factor of urease inhibition/activation potential of the samples. In this bioassay, 25 μL of jack bean urease solution was mixed with phosphate buffer (pH 6.8), the volume of which was 55 μL. Addition of urea (100 mM) followed by incubation at 30 °C with 5 μL of test samples (0.5 mM each) was carried out. This final incubation was done for 15 min in 96-well plates. Next, the addition of phenol reagent (0.005% *w*/*v* sodium nitroprusside and 1% *w*/*v* phenol) was carried out. The volume of phenol reagent taken was 45 μL. In the next step, 70 μL of alkali reagent (0.1% NaOCl and 0.5% *w*/*v* NaOH) was added to each well. After 50 min, the absorbance was noted at 630 nm. Analysis in triplicate for each test sample was carried out in a final volume of 200 μL. The standard inhibitor (positive control) for this urease activity was thiourea [21].

### 3.5. In Silico Studies

#### Selection and Refinement of Protein Structure

**Figure 10:**
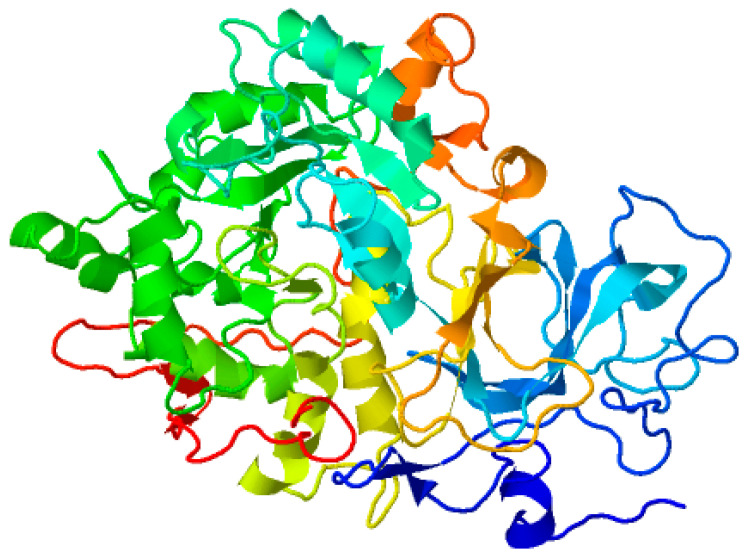
Modeled protein structure optimized using MD simulation.

The amino acid sequence of the target protein urease was obtained from UniProt (www.uniprot.org/uniprot/Q2G2K5 on 7 March 2022), the tertiary structure was designed and the ultimate model was prepared with Modeller 9. This obtained model was further utilized to produce the final full atomic model, and this final model was optimized by MD simulation using AMBER18 (Figure 10).

### 3.6. Validation of Model, Active Site Determination, and Molecular Docking Studies

The model was authorized by examining the phi/psi distributions in the Ramachandran plot attained by the PROCHECK server (https://www.ebi.ac.uk/thornton-srv/software/PROCHECK/ on 7 March 2022). Further, structural quality was examined using ProSA web servers (https://prosa.services.came.sbg.ac.at/prosa.php on 7 March 2022). The prediction of the active site(s) was carried out by means of a site finder tool embedded in MOE-2016. The cavity prediction in a certain protein occurs by means of an active site prediction server that has the residues of the binding site surrounding the cavity at a distance of ∼10 Å. Compounds were drawn using ChemDraw software (https://revvitysignals.com/products/research/chemdraw) and were optimized using Gaussian 09 software at 6–31 G basis set employing B3LYP functional prior to docking. Molecular docking was performed using MOE [22]. Site finder tools from MOE were employed to analyze potential protein binding residues and to create electrostatic surface maps around these residues in order to define docking regions. The MOE tools were utilized to dock synthesized compounds within the target proteins’ specified docking sites. We utilized a triangle algorithm to determine the best-docked molecule poses that were later minimized using the force field refinement technique, and binding energies were taken into consideration while maintaining receptor residues rigid using GB solvation models. The receptor molecule preparation requires the addition of polar hydrogens as well as the merger of nonpolar hydrogens, in accordance with the standard procedure. The top ten ligands were chosen based on their binding energy, together with root-mean-square deviation (RMSD). Finally, all compounds were docked with protein. Drug-like characteristics of the compounds in question were retrieved by the Lipinski filter server.

### 3.7. MD Simulation Studies of Protein–Ligand Complexes as Well as Rescoring of Binding Energies

MD simulation analysis was carried out for the top two (**3a** and **5a**) receptor–ligand complexes. These complexes were selected on the basis of molecular docking studies for confirmation of not only the mode of binding but also the dynamic system stability. Molecular dynamic (MD) simulations were conducted using PMEMD.CUDA from the AMBER 18 suite of programs. To expedite simulation times, an NVIDIA Geforce GTX-1070 Ti graphics card was utilized. The General AMBER Force Field (GAFF) parameters were employed to generate the atomic parameters for each ligand, and Gasteiger charges were assigned to all ligands in the MD simulations.

For each complex structure, periodic boundary conditions were applied, and the system was solvated in a cubic box with TIP3P water molecules extending 12 Å in each direction from the complex model. Additionally, neutralizing Na+ ions were introduced. The cutoff distance for computing unbonded interactions was set to 12 Å. All experimental simulations were conducted under periodic boundary conditions. The AMBER ff14SB force field parameters were employed to characterize the complex. Long-range electrostatic interactions were handled using the particle mesh Ewald (PME) method. To constrain bonds involving hydrogen atoms and maintain temperature control, the SHAKE algorithm and Langevin dynamics were applied. A time step of 2 fs was utilized, and trajectory data were recorded every 0.2 ps. The system’s temperature was gradually raised from 0 to 310.15 K over 100 ps of NVT dynamics, followed by 10 ns of NPT equilibration at 310.15 K and 1 atm pressure. Finally, a total of 100 ns of production phase MD simulations were performed to collect properties [23]. Trajectory analyses, including root-mean-square deviation and fluctuation, were conducted using the CPPTRAJ module within the Amber 18 program. To calculate the binding free energy for each simulation complex, the Amber molecular mechanics Poisson–Boltzmann surface area (MM-PBSA) method was employed. A total of 300 structural frames were selected from the 80 ns trajectory data. Subsequently, 1000 snapshots were extracted from the trajectory data for binding free energy calculations using the MM-PBSA method. The grid size utilized in the Poisson–Boltzmann calculations within MM-PBSA was set to 0.5 Å [24].

### 3.8. In Vivo Pharmacology

#### Experimental Animals

The rats used in this study were Swiss albino mice (male and female; weight range: 30–35 g) The animals were acquired from the National Institute of Health, Islamabad, Pakistan. Written approval was obtained from the departmental ethics committee (DREC/20). Also, the approved animal house was used to preserve the animals [25]. All of the standard ethical guidelines were followed during the course of this study [26].

Pylorus ligation activity [27], ethanol-induced ulcer [28], aspirin-induced ulcer [29], histamine-induced ulcer [30] and H^+^–K^+^ ATPase assay [31] studies were conducted as per standard operating procedures with respective standards.

## 4. Conclusions

A total of 1 reported and 10 new derivatives of chromene were synthesized with enhanced/modified biological activity. The antibacterial activity hints at the antibacterial potential of synthesized 4-aminocoumarin derivatives. The present work also presents the in silico inhibitory potential of synthesized compounds against urease enzyme. Out of ten substituted chromenone Schiff bases, **3a** and **5a** showed significantly increased binding potential at the urease active site, as inferred from molecular docking and molecular dynamics simulation study. Compounds **3a** and **5a** interacted with Ni ion via Ni-O bonding with three key amino acids (His492, His 593, and Asp494) facilitated by strong H-bonds/hydrophobic interactions that are critical for the activity of urease receptors. These newly synthesized compounds were also tested for in vitro urease inhibition potential. This investigation yields a few crucial findings. First, two compounds were identified that represent scaffolds that are different from those previously recognized; they are all significantly smaller than most of the known urease inhibitors, giving them comparatively good ligand efficiencies. Furthermore, compounds **3a** and **5a** IC_50_ values of 0.412 µM (64.0% inhibition) and 0.322 µM (77.7% inhibition), respectively, together with their good physical qualities, place them in the lead-like range of compounds that might be optimized as bioactive molecules for the urease enzyme. Additionally, the rat model with pyloric ligation-induced ulcers exhibited elevated acidity levels, including total acidity and free acidity, in Group I compared to Group V, which indicated the severity of ulceration. However, the introduction of compound **5a** significantly reduced these acidity levels, suggesting its potential effectiveness in mitigating ulceration induced by ethanol. Furthermore, compound **5a** demonstrated a substantial reduction in lipid peroxidation and a significant increase in gastric pH. It also led to a remarkable decrease in the volume of gastric content. These findings collectively support the potential therapeutic role of compound **5a** in reducing ethanol-induced ulcers in the rat model. Moreover, compound **5a** displayed a noteworthy inhibitory effect on gastric ATPase, with an IC_50_ value of 30 μg/mL, comparable to the positive control omeprazole (IC_50_ of 22 μg/mL). This suggests that compound **5a** may act as a gastric ATPase inhibitor, further highlighting its potential as a treatment option for gastric ulcers. Overall, the comprehensive results presented in the study suggest that compound **5a** holds promise as a therapeutic agent for the management of ethanol-induced ulcers, with potential benefits in reducing acidity, lipid peroxidation, and gastric ATPase activity. Further investigations and clinical studies are warranted to fully explore its therapeutic efficacy and safety for clinical application.

## Figures and Tables

**Table 1:** Optimization conditions for the first step of the reaction.

1st Reactant (Equiv.)	2nd Reactant (Equiv.)	Solvents	Temp. (°C)	Time (h)	Results
1	1	Ethoxyethanol	170	48	Too many side products are present
1	2	Glacial acetic acid	180	72	Too many side products are present
1	2	Solvent-free	130	05	No reaction
1	5	Solvent-free	170	14–15	70% yield

**Table 2:** Optimization conditions for the second step of reaction.

1st Reactant (Equiv.)	2nd Reactant (Equiv.)	Solvents	Temp. (°C)	Time (h)	Results
1	1	Ethanol	170	48	10% yield
1	1.5	Ethanol + glacial acetic acid (3–4 drops)	170	72	10% yield
1	1.5	Methanol + glacial acetic acid (3–4 drops)	170	72	15% yield
1	1	Analytical grade methanol	170	36–82 Continuous reflux	65–75% yield

**Table 3:** Inhibition of synthesized compounds and their IC_50_ value along with standard deviation/error of the mean.

Compound No.	% Inhibition ± STD	IC_50_ ± SEM (µM)
**3a**	64.0 ± 0.42	0.412 ± 0.10
**5a**	77.7 ± 0.64	0.322 ± 0.13
**7a**	60.2 ± 0.32	0.112 ± 0.26
Thiourea	82.0 ± 0.15	0.140 ± 0.22

**Table 4:** Binding energies and metal-oxygen binding of the compounds in the active site of urease enzyme.

Compounds ID	Binding Kcal/mol	Ni Binding
**2a**	−3.547	Absent
**3a**	−8.677	Present (Ni-O 2.3 Å)
**4a**	−5.180	Present (Ni-O 2.5 Å)
**5a**	−7.443	Present (Ni-O 2.4 Å)
**6a**	−5.892	Present (Ni-O 2.7 Å)
**7a**	−6.834	Present (Ni-O 2.3 Å)
**8a**	−4.131	Absent
**9a**	−6.967	Present (Ni-O 2.6 Å)

## Data Availability

Data is contained within the article and supplementary material.

## Supplementary Materials

The following supporting information can be downloaded at: https://www.mdpi.com/article/10.3390/ph16111552/s1, Figure S1. Spectral Data of all compounds.
