# Supplementary material for: Novel Coumarin Derivatives as Potential Urease Inhibitors for Kidney Stone Prevention and Antiulcer Therapy: From Synthesis to In Vivo Evaluation
Source: Pharmaceuticals (Basel). 2023 Nov 2;16(11):1552. doi: 10.3390/ph16111552 (PMC10675132; doi:10.3390/ph16111552)
Supplement: Supplementary file 1 [file pharmaceuticals-16-01552-s001.zip › pharmaceuticals-2626104-supplementary.pdf]

Novel Coumarin Derivatives as Potential Urease Inhibitors for Kidney Stone Prevention and Antiulcer Therapy: From Synthesis to In Vivo Evaluation

Kiran Shahzadi [1,2], Syed Majid Bukhari [1], Asma Zaidi [1,*], Tanveer A. Wani [3], Muhammad Saeed Jan [4], Seema Zargar [5], Umer Rashid [1], Umar Farooq [1,6], Aneela Khushal [1] and Sara Khan [1,*]

[1] Department of Chemistry, COMSATS University Islamabad, Abbottabad Campus, Abbottabad 22060, KPK, Pakistan; kiranshahzadi12@gmail.com (K.S.); majidbukhari@cuiatd.edu.pk (S.M.B.); umerrashid@cuiatd.edu.pk (U.R.); umarf@cuiatd.edu.pk (U.F.); aneelakhan460@gmail.com (A.K.)

[2] School of Chemistry and Chemical Engineering Technology, Beijing Institute of Technology,
Beijing 100811, China

[3] Department of Pharmaceutical Chemistry, College of Pharmacy, King Saud University, P.O. Box 2457,
Riyadh 11451, Saudi Arabia; twani@ksu.edu.sa

[4] Department of Pharmacy, The Professional Institute of Health Sciences, Mardan 23200, KPK, Pakistan

[5] Department of Biochemistry, College of Science, King Saud University,
P.O. Box 22452, Riyadh 11451, Saudi Arabia; szargar@ksu.edu.sa

[6] Beijing National Laboratory for Molecular Sciences, State Key Laboratory of Molecular Reaction Dynamics, Institute of Chemistry, Chinese Academy of Sciences, Beijing 100190, China

* Correspondence: asmazaidi@cuiatd.edu.pk (A.Z.); sarakhan@cuiatd.edu.pk (S.K.)

Figure S1: Spectral Data of all compounds

Compound 2a

NMR

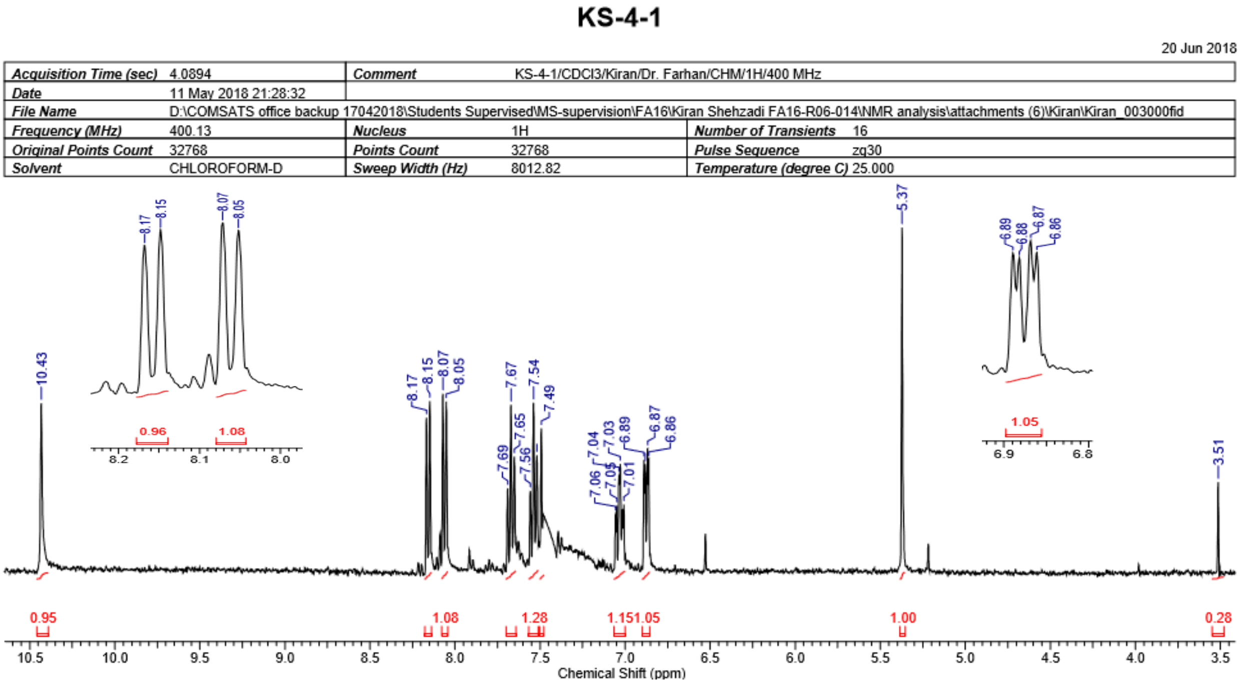

FT-IR

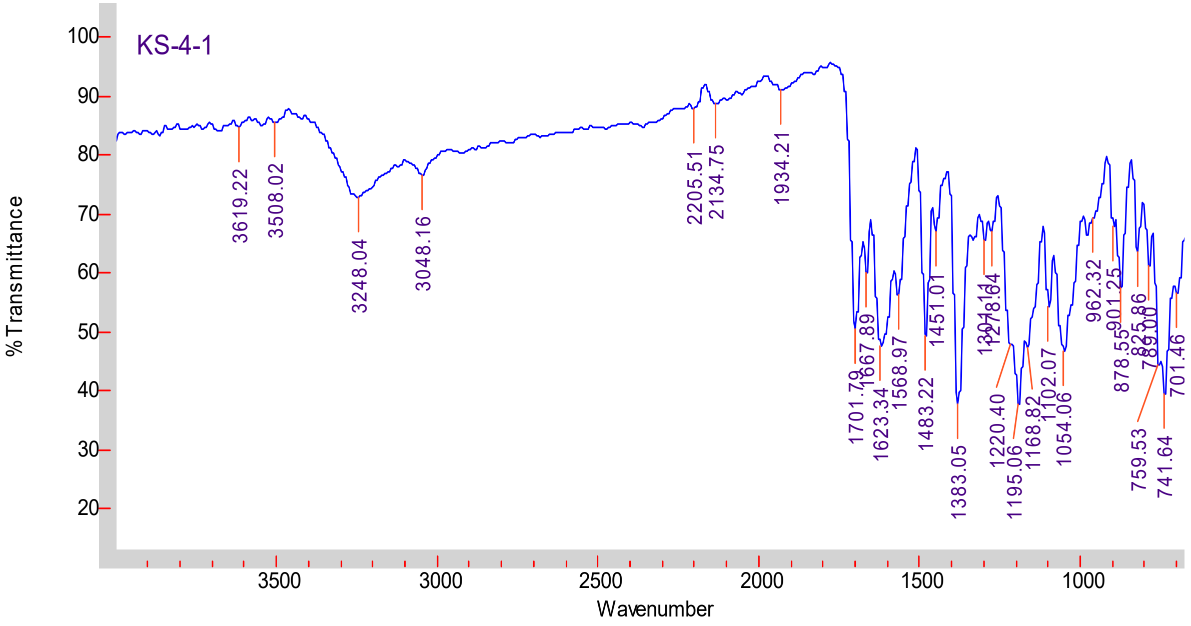

Mass spectrum

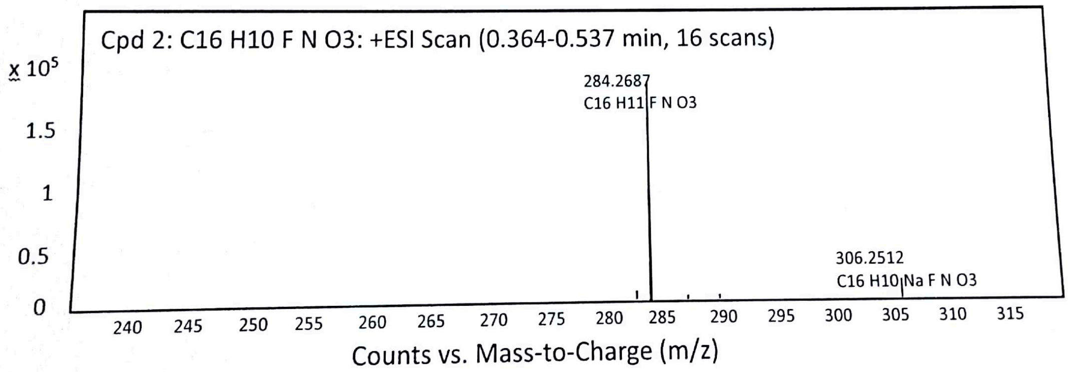

Compound 3a

NMR

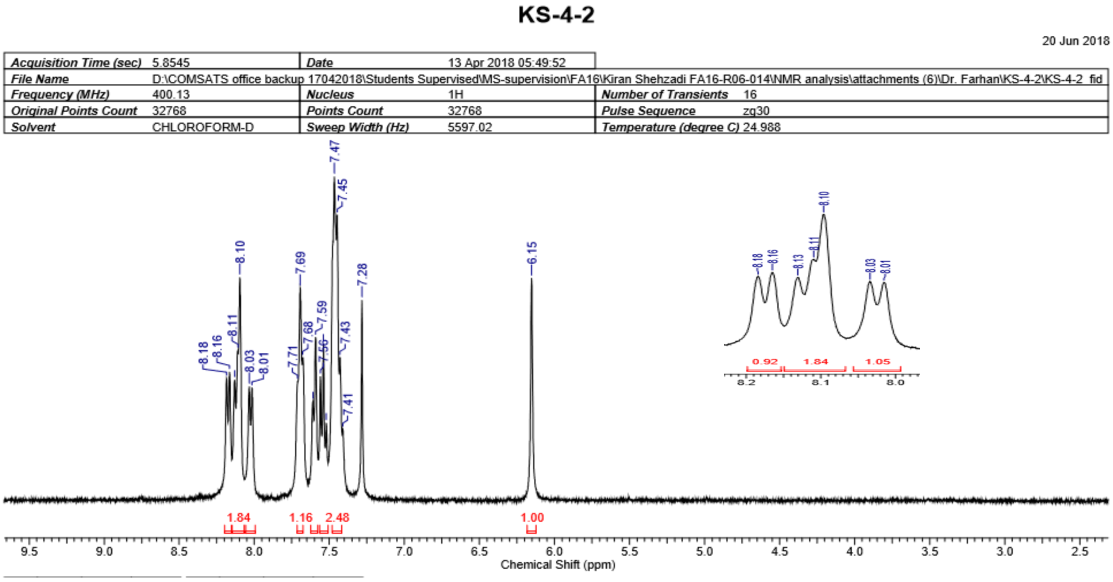

FT-IR

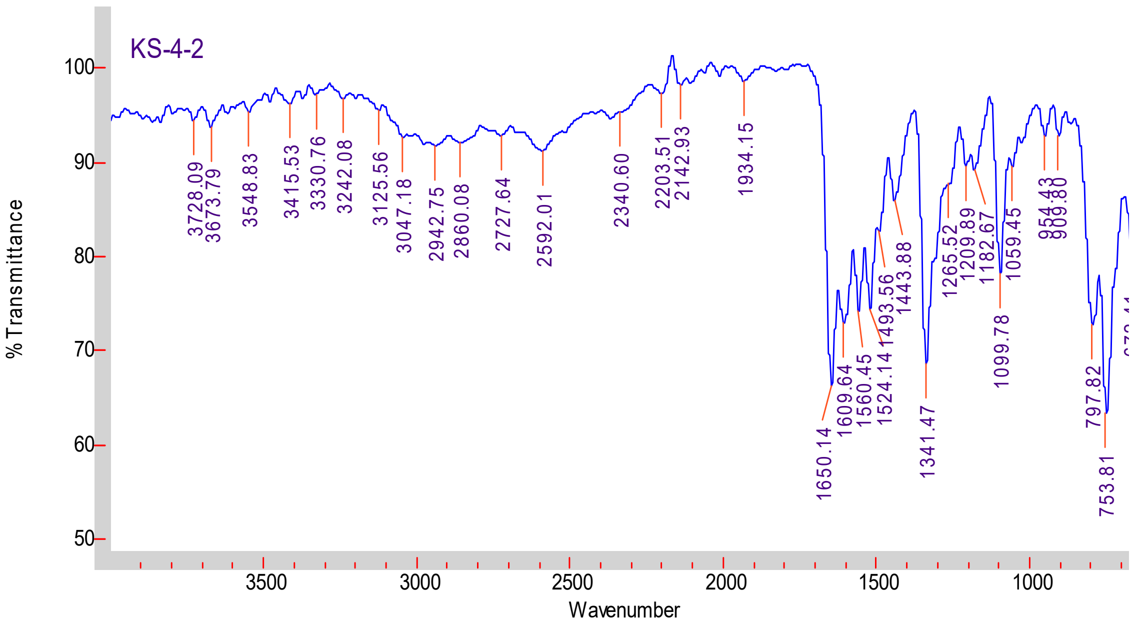

Mass Spectrum

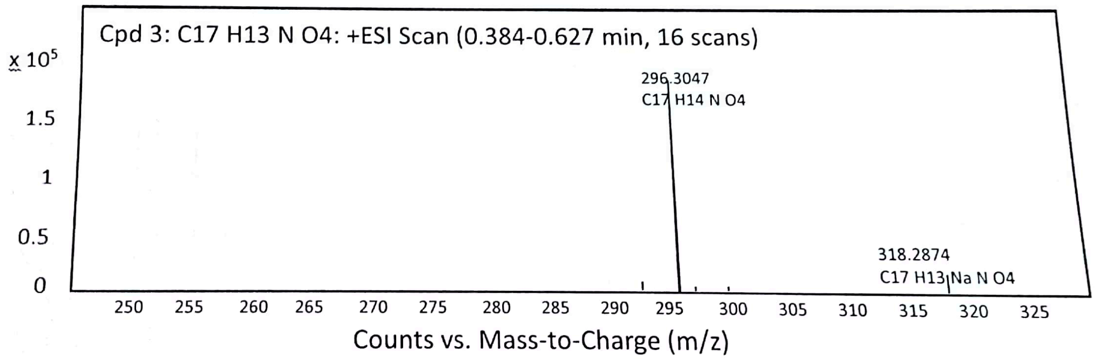

HPLC Chromatogram

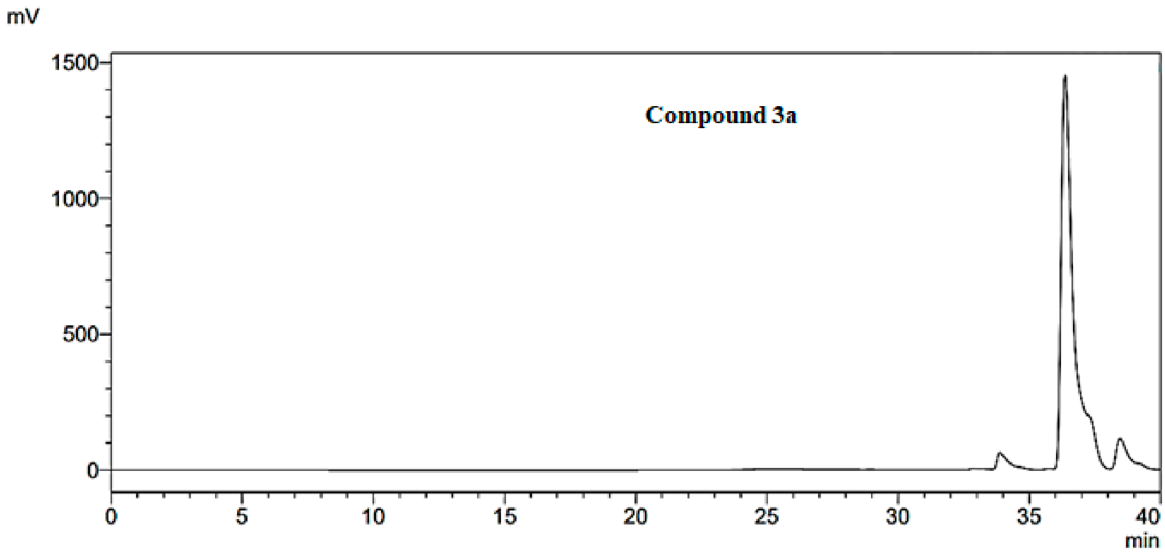

Compound 4a

NMR

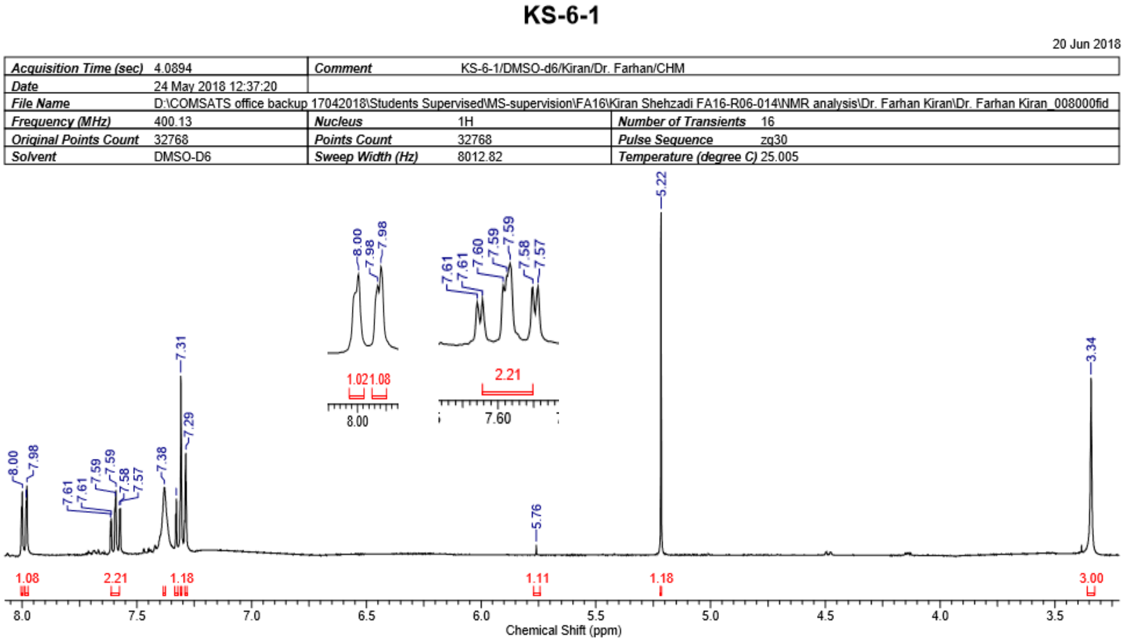

FT-IR

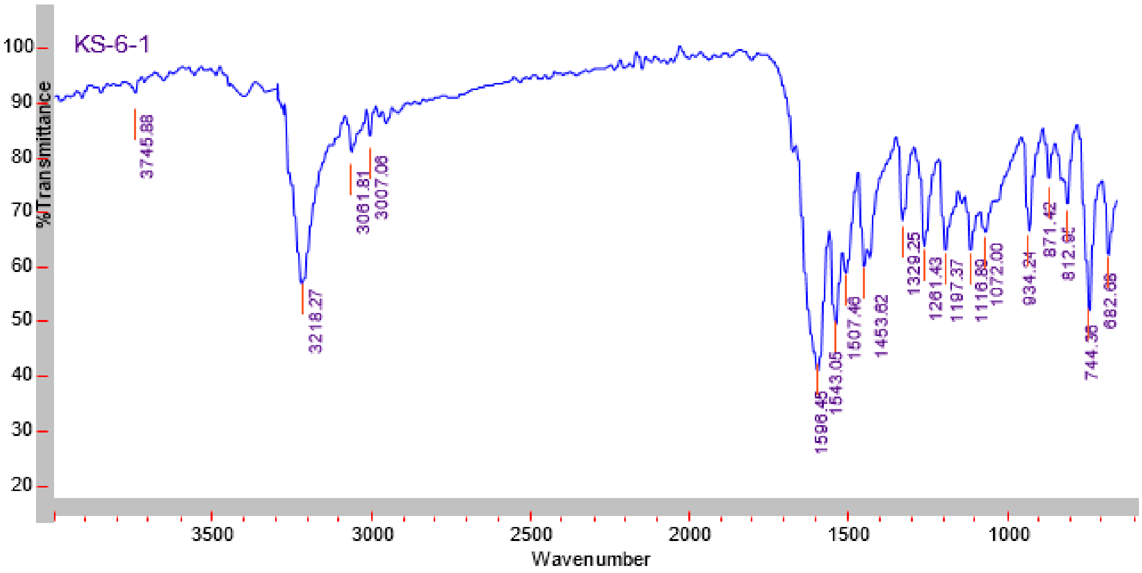

Compound 5a

NMR

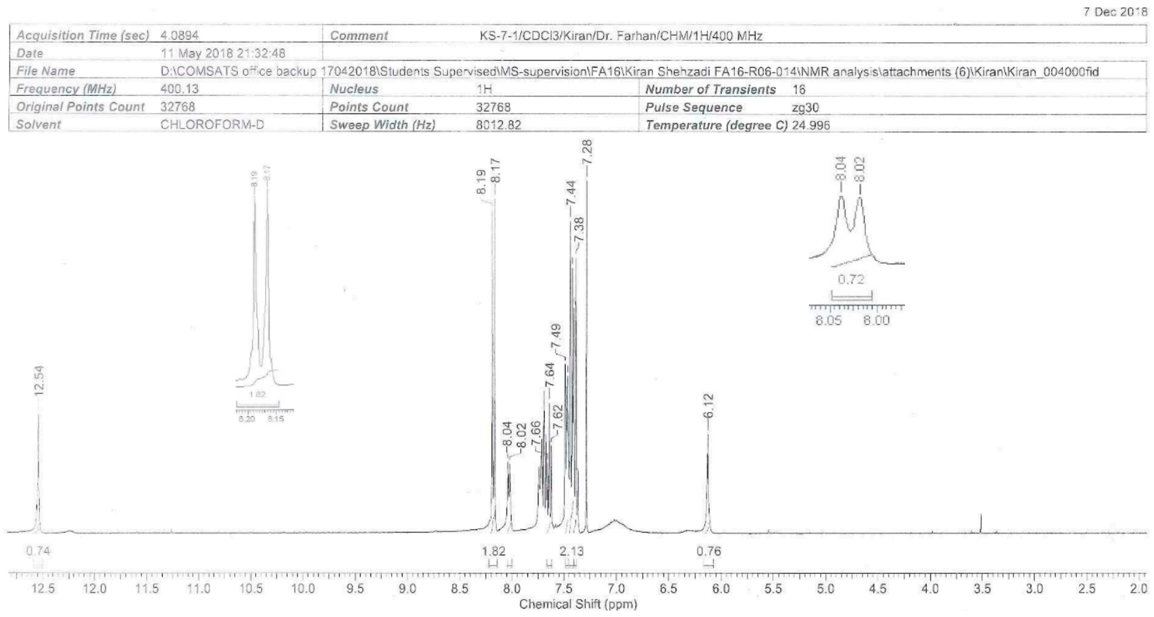

FT-IR

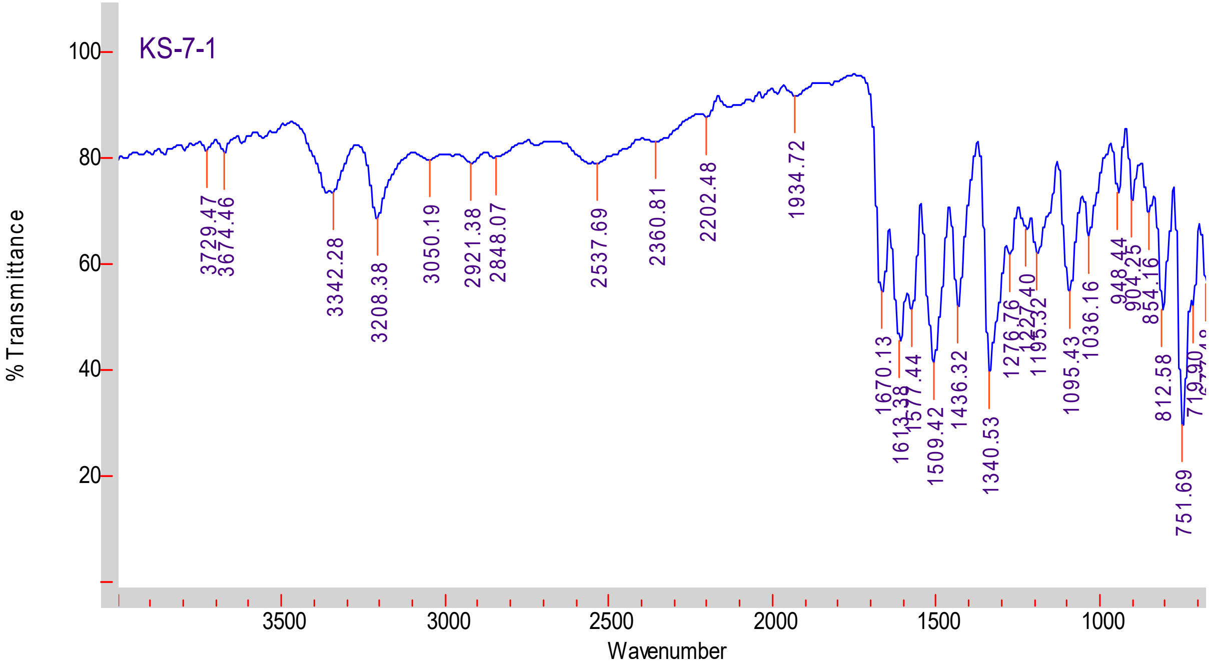

HPLC Chromatogram

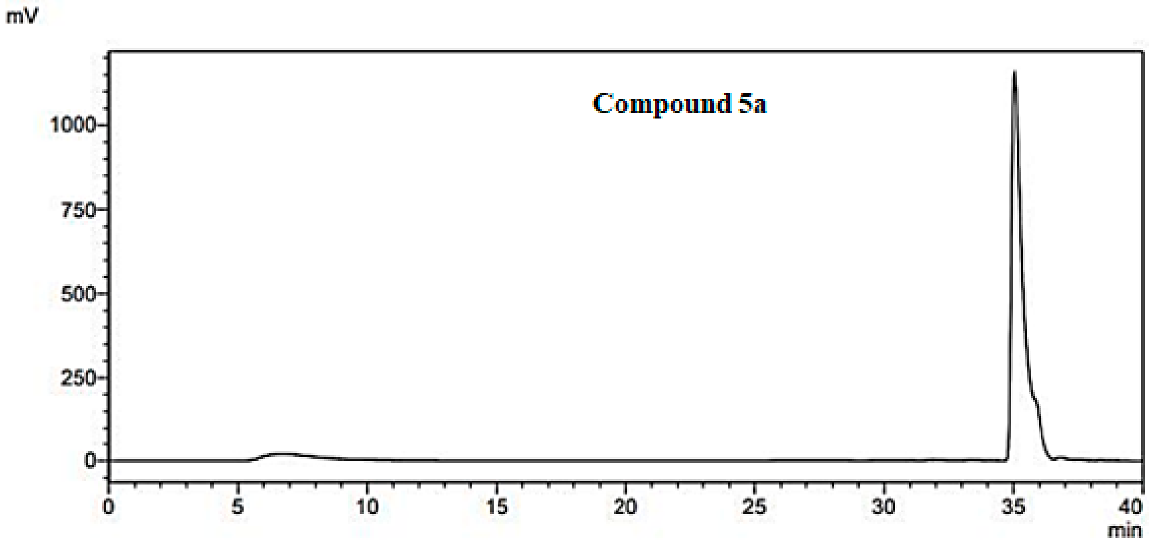

Compound 6a

NMR

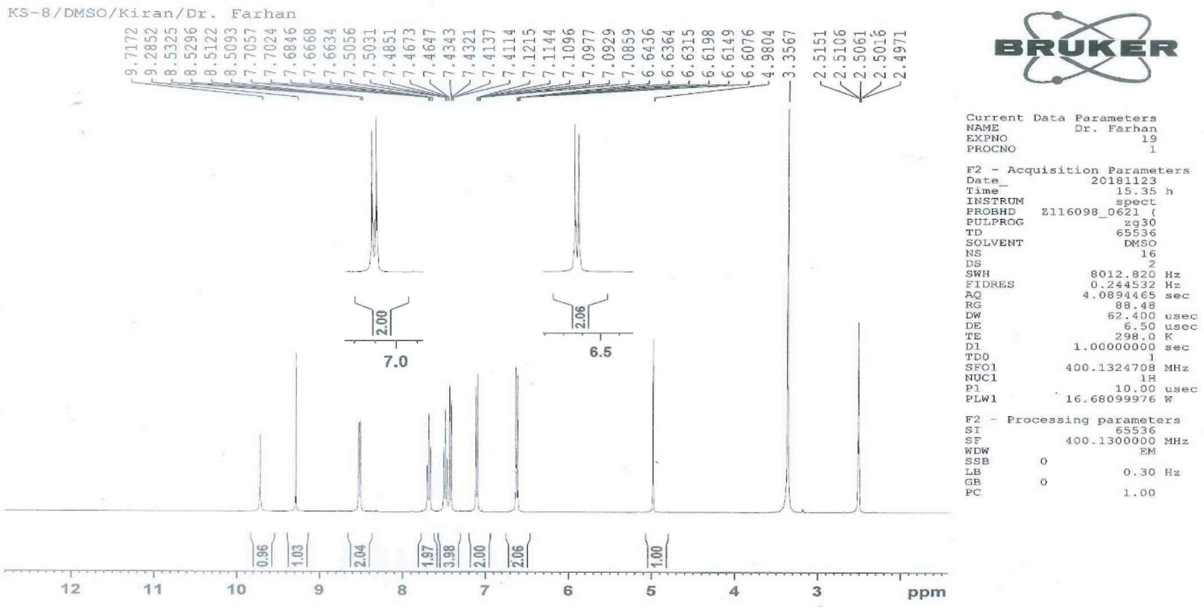

FT-IR

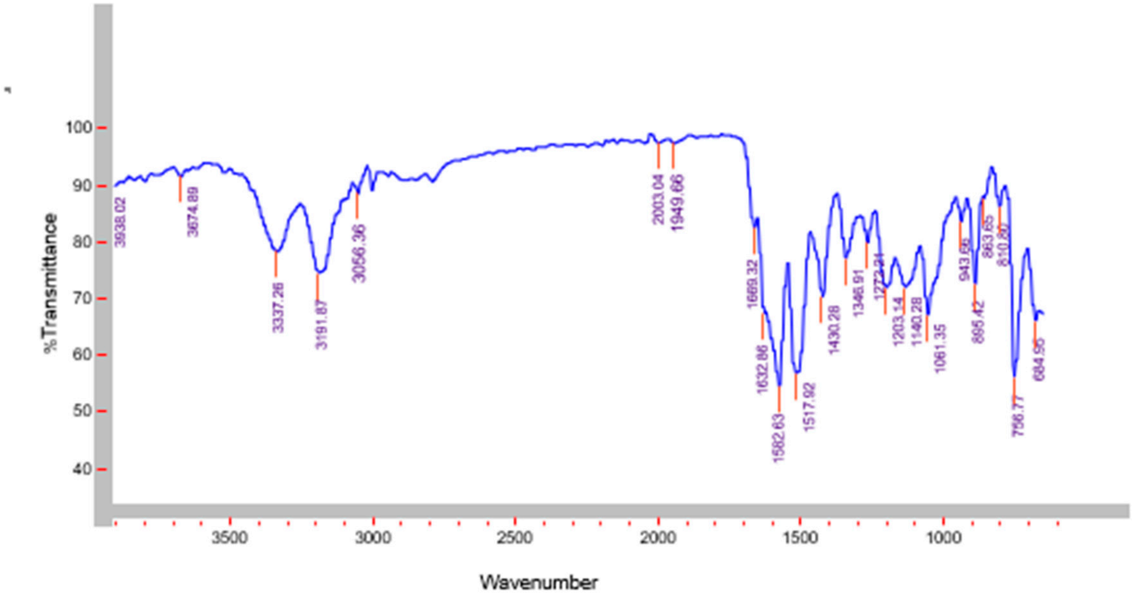

Compound 7a

NMR

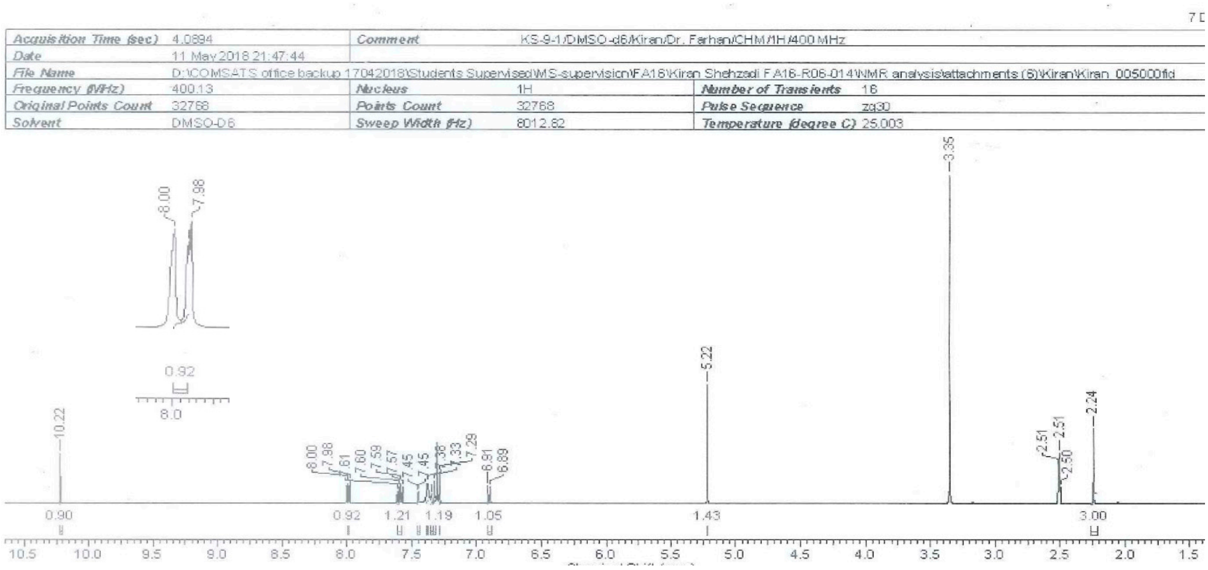

FT-IR

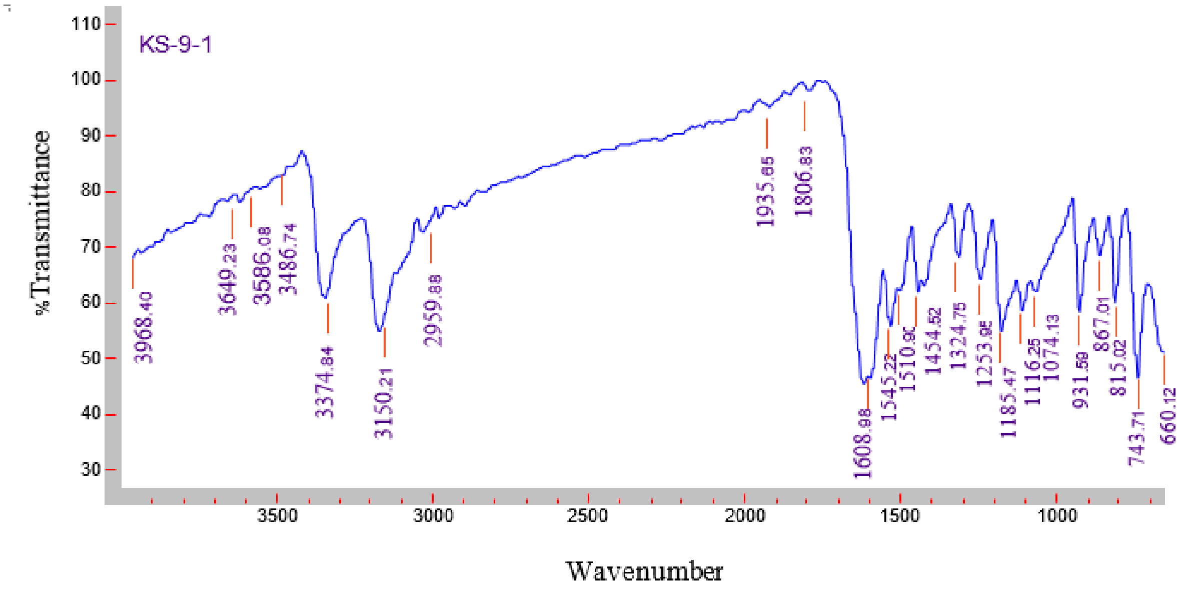

Compound 8a

NMR

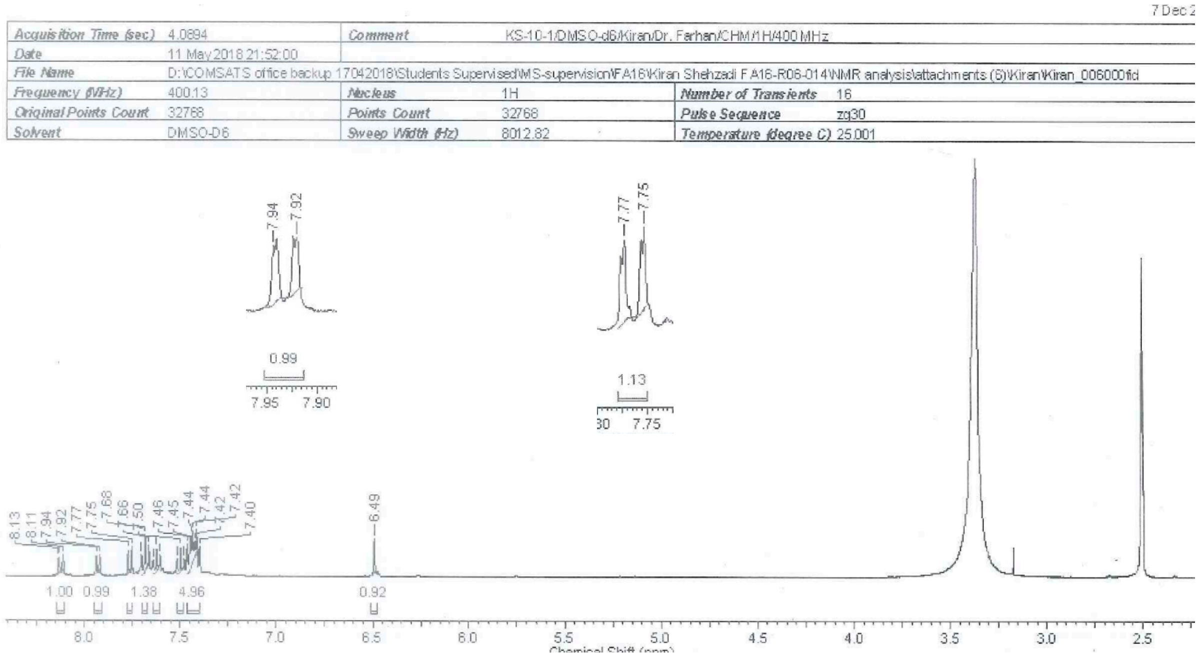

FT-IR

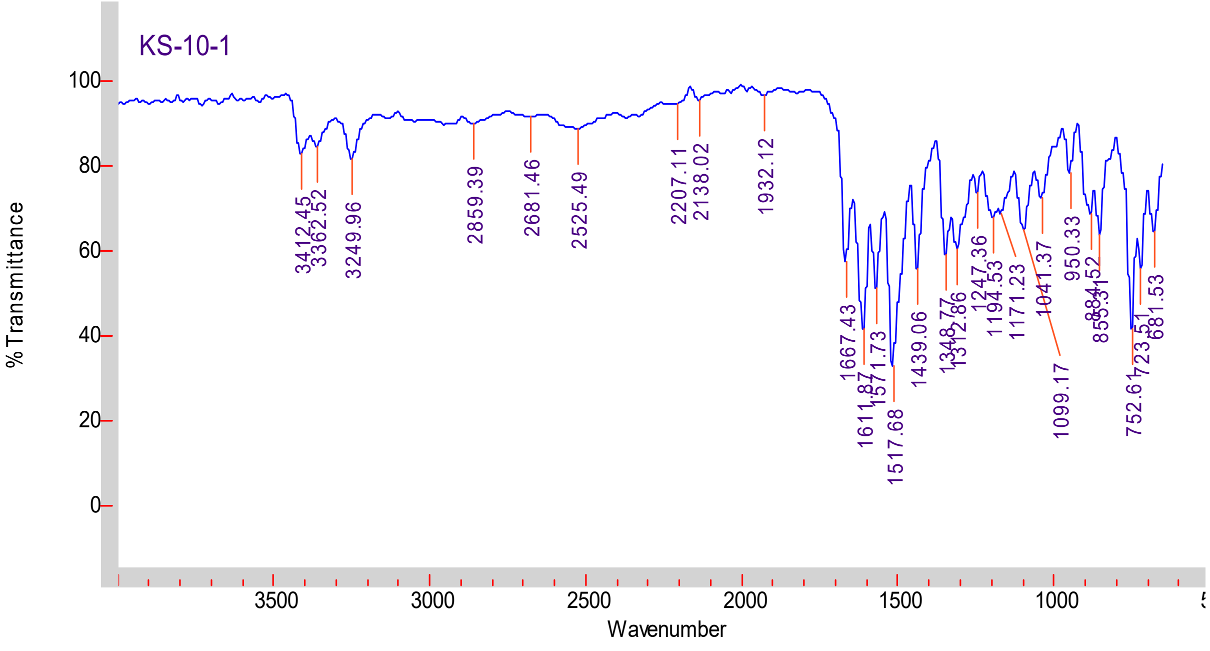

Compound 9a

NMR

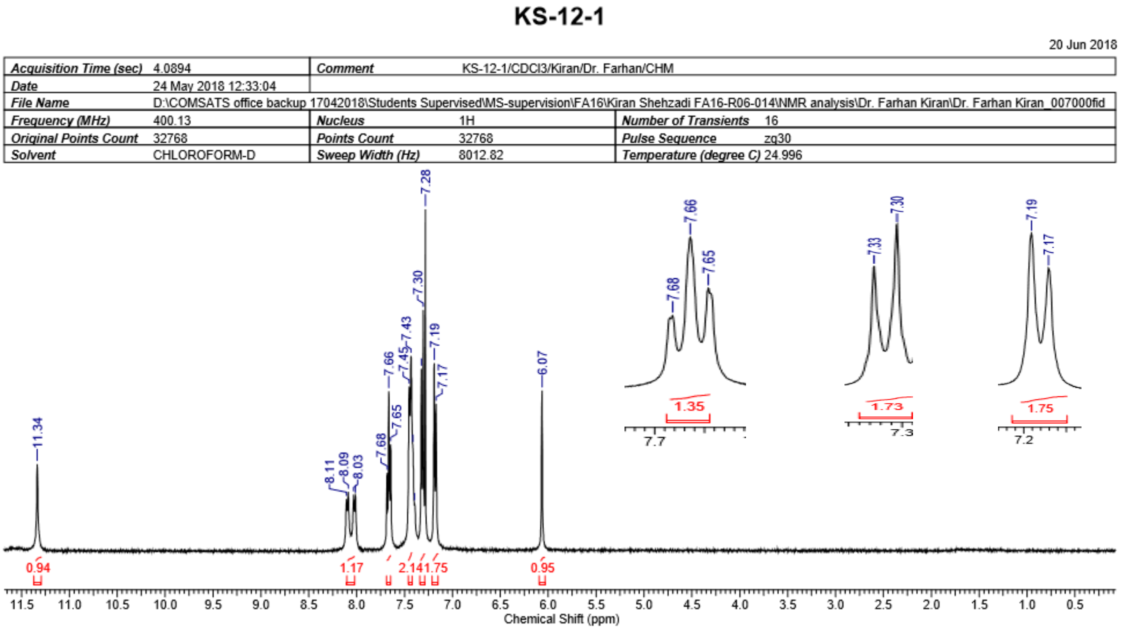

FT-IR

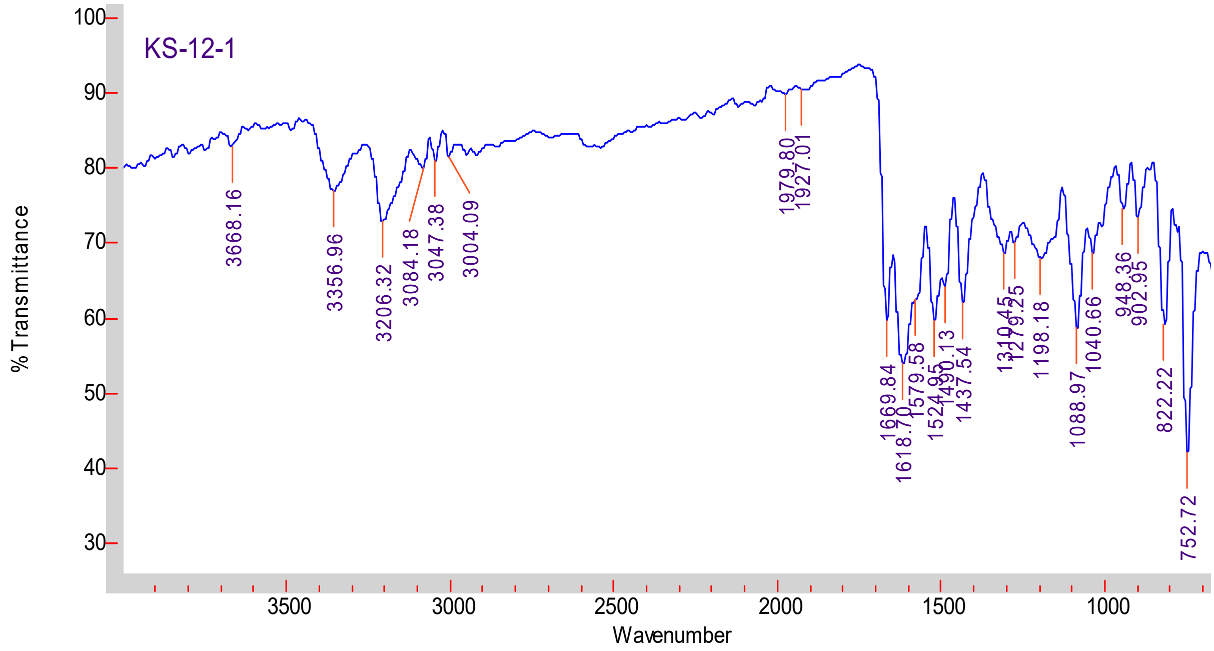

Compound 10a

NMR

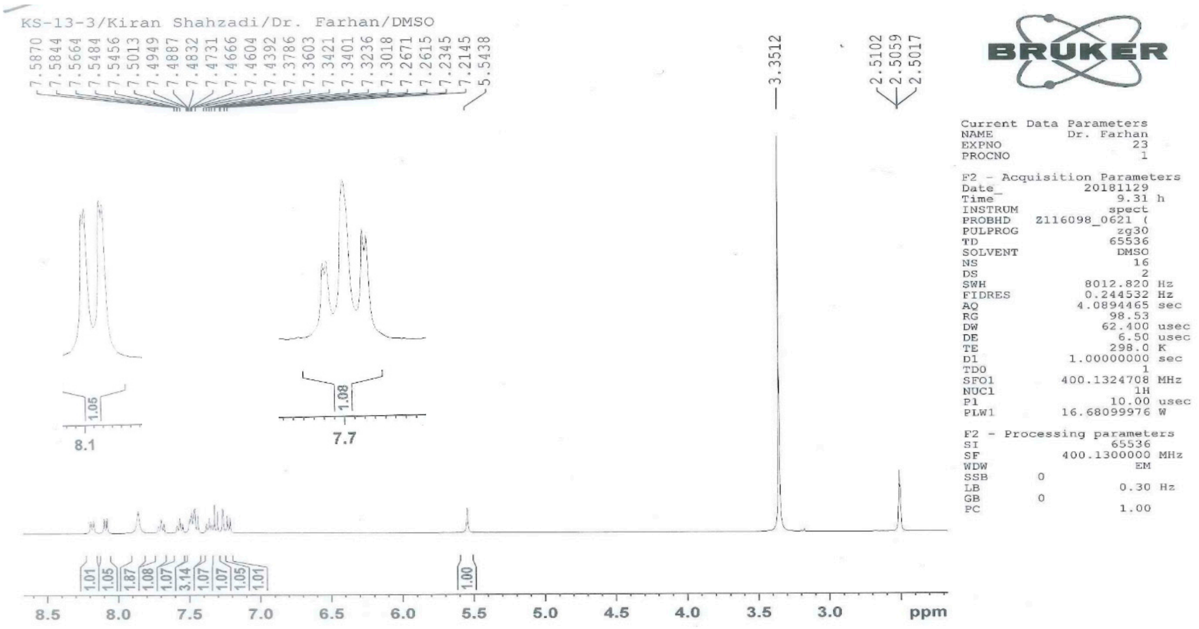

FT-IR

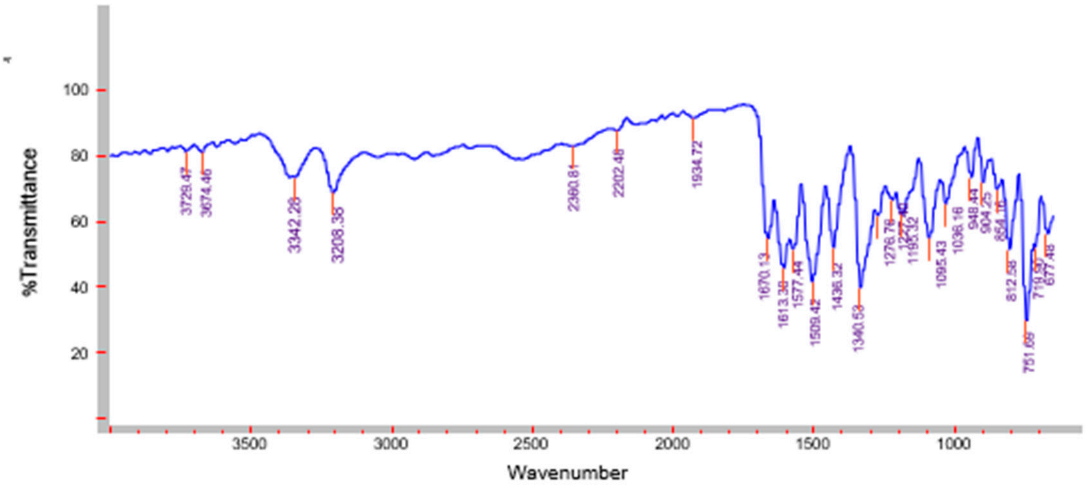

Compound 11a

NMR

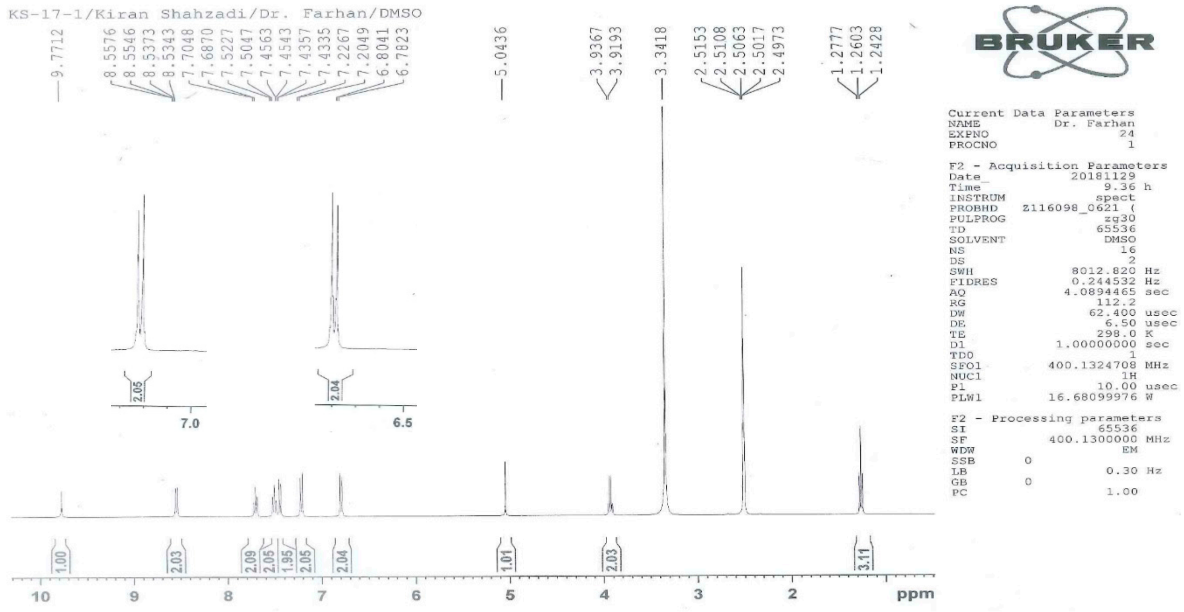

FT-IR

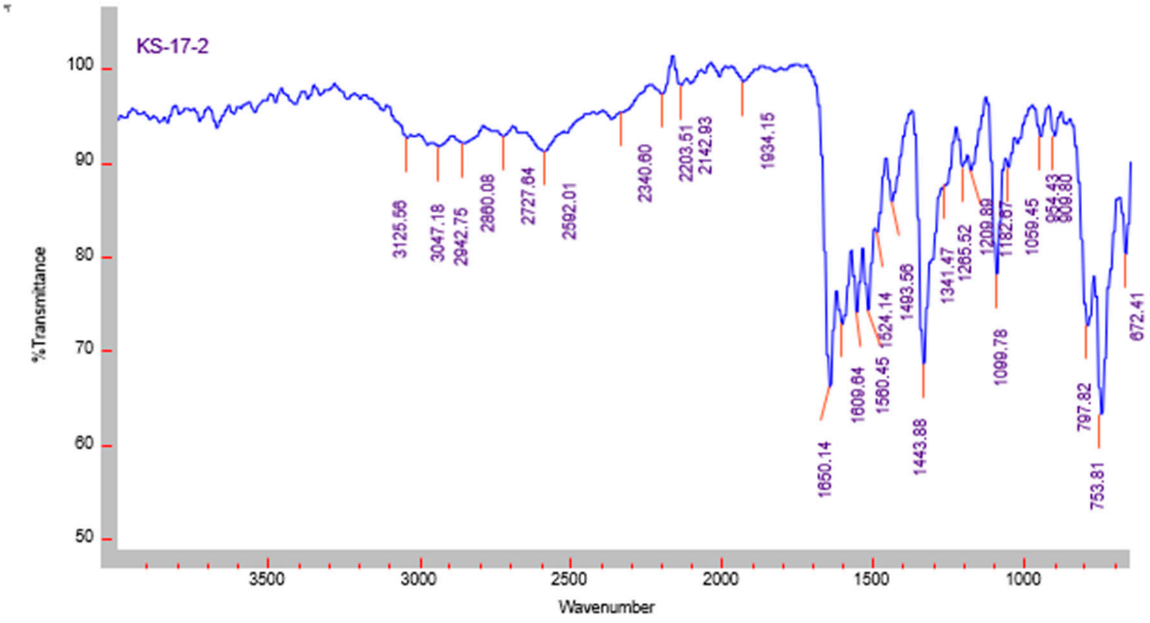

KS-17-2
%Transmittance
100
90
80
70
60
50
3500
3000
2500
2000
1500
1000
Wavenumber
3125.56
3047.18
2942.75
2860.08
2727.64
2592.01
2340.60
2203.51
2142.93
1934.15
1650.14
1609.64
1560.45
1524.14
1493.56
1443.88
1341.47
1285.52
1209.89
1182.67
1099.78
1059.45
954.43
909.80
797.82
753.81
672.41
